# Deacetylation of SRF catalyzed by SIRT7 protects against abdominal aortic aneurysm formation

**DOI:** 10.1038/s12276-026-01787-z

**Published:** 2026-07-08

**Authors:** Yacheng Xiong, Jing Chen, Weiting Lu, Yu Liu, Chang Sheng, Shuai Liu, Baohui Xu, Yongqing Li, Baihong Pan, Wei Wang

**Affiliations:** 1 Department of General and Vascular Surgery, Xiangya Hospital, Central South University, Changsha, China; 2 National Clinical Research Center for Geriatric Disorders, Xiangya Hospital, Central South University, Changsha, China; 3 Department of Surgery, Stanford University School of Medicine, Stanford, CA USA; 4 Department of Surgery, University of Michigan Medical School, Ann Arbor, MI USA; 5 Clinical Research Center for Vascular Intervention in Hunan Province, Xiangya Hospital, Central South University, Changsha, China

**Keywords:** Acetylation, Aneurysm, Ubiquitylation

## Abstract

Abdominal aortic aneurysm (AAA) is a life-threatening cardiovascular disease for which no effective pharmacological treatments exist. Histone deacetylases (HDACs) have emerged as critical regulators of cardiovascular pathophysiology; however, the contributions of individual HDAC isoforms to AAA formation remain largely undefined. Here, we identified sirtuin 7 (SIRT7) as the most markedly downregulation isoform among 18 HDAC family members in human AAA tissues. Global and vascular smooth muscle cell (VSMC)-specific *Sirt7* knockout exacerbated AAA formation, whereas *SIRT7* overexpression attenuated aneurysm progression. Mechanistically, SIRT7 deacetylates serum response factor (SRF) at lysine 154, thereby preventing SRF nuclear-to-cytoplasmic translocation and ubiquitin-mediated degradation and preserving the VSMC contractile phenotype. Consequently, SIRT7 deficiency enhances SRF acetylation, accelerates its degradation, and promotes VSMC phenotypic switching, ultimately facilitating AAA progression. Pharmacological activation of SIRT7 with the NAD⁺ precursor nicotinamide mononucleotide attenuated aortic dilation. Collectively, our findings reveal a crucial role for SIRT7-mediated SRF-K154 deacetylation in limiting AAA progression and suggest that nicotinamide mononucleotide supplementation may represent a promising therapeutic strategy for AAA.

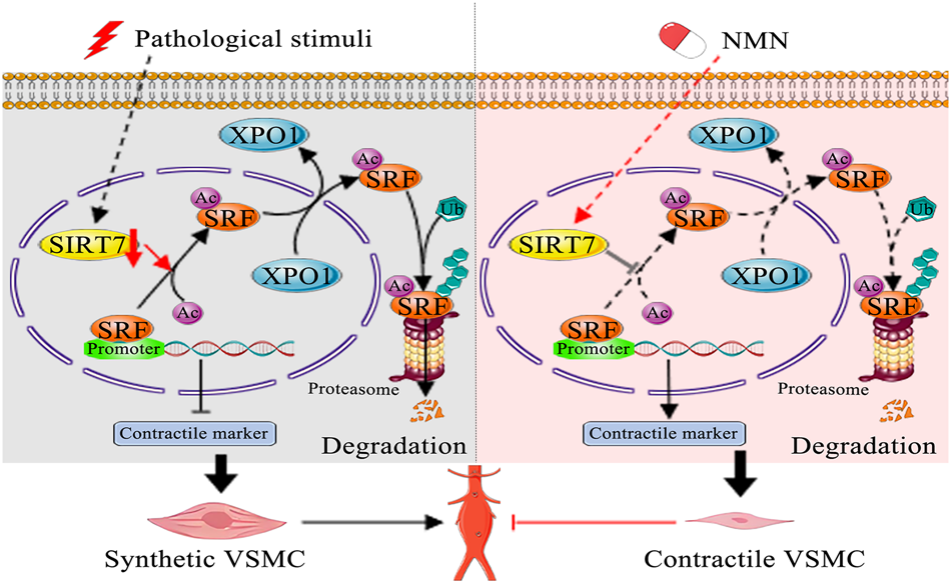

## Targeting SIRT7 offers new hope for aortic aneurysms

Abdominal aortic aneurysm (AAA) is a severe vascular condition with a high mortality rate owing to rupture. This study identifies sirtuin 7 (SIRT7), a member of the histone deacetylase family, as a protective factor against AAA by maintaining vascular smooth muscle cell (VSMC) contractility through deacetylation of the serum response factor (SRF) at lysine 154. Researchers used human and murine models to show that SIRT7 is downregulated in AAA, leading to pathological SRF acetylation, nuclear export, and degradation, which promotes VSMC phenotypic switching and aneurysm development. Importantly, activating SIRT7 with nicotinamide mononucleotide (NMN) significantly reduced aneurysm progression in vivo. These findings suggest that targeting SIRT7-mediated SRF deacetylation could be a promising therapeutic strategy for AAA, with NMN offering a potential non-surgical treatment avenue. Future research should focus on developing SIRT7-specific activators to enhance therapeutic efficacy and safety.

This summary was initially drafted using artificial intelligence, then revised and fact-checked by the author.

## Introduction

Abdominal aortic aneurysm (AAA) is a life-threatening vascular disease characterized by progressive dilation of abdominal aorta and a high risk of catastrophic rupture. The incidence of AAA continues to rise with population aging and a rupture mortality rate of up to 80% (refs. ^1,2^). Despite advances in surgical techniques, there are currently no effective pharmacological therapies proven to prevent or delay AAA progression^3^. The pathogenesis of AAA involves multiple mechanisms, including inflammatory infiltration, smooth muscle dysfunction, extracellular matrix degradation, and endothelial oxidative stress injury^4^. Accumulating evidence indicates that vascular smooth muscle cell (VSMC) dysfunction has an important role in the development of aortic aneurysms^5^. Therefore, defining the molecular mechanisms regulating VSMC homeostasis is critical for advancing theoretic strategies, which may provide important insights for AAA treatment.

The histone deacetylase (HDAC) family, a group of enzymes that regulate post-translational modification of proteins, regulates post-translational acetylation of histone and non-histone proteins and has fundamental roles in gene transcriptional regulation, cell survival, and disease progression^6^. Several members of the HDAC family have been implicated in the pathological and physiological processes of cardiovascular diseases^7^. Recent studies have demonstrated that the expression levels of HDAC1, HDAC2, HDAC4, HDAC5, and HDAC7 are elevated in AAA^8–10^, and that pharmacological inhibition of HDAC activity can attenuate aneurysm progression^8,9,11–13^. Interestingly, Zhang et al.^14^ reported that treatment with a pan-HDAC inhibitor aggravated aneurysm formation, highlighting a paradoxical and incompletely understood phenomenon. Among the HDAC family, SIRT1 and SIRT6 have been the most extensively investigated in AAA, primarily modulating inflammation, oxidative stress, and cellular senescence through histone deacetylation^15–18^. Nevertheless, the expression patterns of all HDAC family members in human AAA have not been systematically investigated, nor have there been any reports on the involvement of non-histone post-translational modifications of sirtuin proteins in AAA. To date, a comprehensive analysis of all HDAC family members in human AAA tissues has been lacking, and little is known about the contributions of non-histone deacetylation targets of sirtuins to AAA pathobiology. Therefore, elucidating the expression profiles and molecular mechanisms of HDACs may provide novel insights for the treatment of AAA.

Sirtuin 7 (SIRT7), a member of the silent information regulator 2 protein (sirtuin) family, is an NAD^+^-dependent deacetylase that has garnered considerable attention owing to its central roles in anti-apoptotic, anti-inflammatory, and anti-aging processes^19^. Emerging evidence suggests that SIRT7 is involved in various age-related diseases, including cancer, metabolic disorders, and cardiovascular diseases^20^. Specifically, SIRT7 deficiency has been shown to promote cardiomyocyte apoptosis and exacerbate cardiac inflammation, leading to cardiomyopathy^21^. Moreover, SIRT7 has been shown to regulate smooth muscle remodeling in the airway^22^. However, the role of SIRT7 in AAA and its underlying mechanisms remain unexplored.

Here, we identify that SIRT7 expression is significantly downregulated in human AAA. Our findings demonstrate that SIRT7 exerts a protective role against aneurysm formation by deacetylating serum response factor (SRF) and suppressing VSMC phenotypic switching. Importantly, this study reveals that SIRT7-mediated SRF-K154 deacetylation is critical for SRF nuclear localization, protein stability, and transcriptional activity. Loss of SIRT7 results in pathological SRF acetylation, promoting nuclear export and ubiquitin-mediated degradation, thereby driving VSMC phenotypic switching and aneurysm development. These results suggest that pharmacological activation of SIRT7 may represent a promising therapeutic strategy for AAA. Finally, we demonstrate that activation of SIRT7 with the NAD⁺ precursor nicotinamide mononucleotide (NMN) significantly attenuates aneurysm progression in vivo, identifying a previously unrecognized and potentially druggable pathway for AAA therapy.

## Experimental model and study participant details

### Reagents

The key reagents used in this experiment can be obtained in Supplementary Table 1.

### Human studies

All human studies were approved by the Ethics Committee of Central South University Xiangya Hospital (Approval No. 201803481) in accordance with the Declaration of Helsinki principles. Patients with AAA were recruited in Xiangya Hospital, Central South University (Changsha, China) from July 2018 to September 2024. AAA was confirmed by CT angiography according to the 2018 American Society for Vascular Surgery guidelines for the diagnosis and treatment of AAA. The AAA samples were obtained from the patient receiving open aneurysm repair. The control tissue sample was derived from adjacent relatively normal abdominal aorta from the same patient. The clinical characteristics of patients can be obtained in Supplementary Tables 2 and 3. All included subjects provided written informed consent.

### Animal studies

All animal studies were approved by the Animal Laboratory Center Ethics Committee of Xiangya Hospital (Approval No. 2020309026) and all animal procedures adhered to the US National Institutes of Health’s Guide for the Care and Use of Laboratory Animals. Sirt7 knockout mice (*Sirt7*^*+/*^^*−*^), Sirt7 flox mice (*Sirt7*^*flox/+*^), and *Tagln-Cre* mice were purchased from Cyagen Biosciences, Inc. (Suzhou, China). ApoE knockout mice (*ApoE*^*−/−*^) and wild-type (WT) mice were purchased from Hunan SJA Laboratory Animal Co., Ltd (Changsha, China). VSMC-specific Sirt7 knockout mice (*Sirt7*^Δ*SMC*^) were generated by crossbreeding *Sirt7*^*flox/+*^ with *Tagln-Cre* mice. *Sirt7*^*+/*^^*−*^ and *Sirt7*^Δ*SMC*^ were crossed with *ApoE*^*−/−*^ to establish *ApoE*^*−/−*^*Sirt7*^*−/−*^ and *ApoE*^*−/−*^*Sirt7*^Δ*SMC*^ mice. All animals were housed in SPF conditions at 23 ± 1 °C with 50–60% humidity in a 12-h light and dark cycle. The angiotensin II (AngII)-induced or calcium chloride (CaCl_2_)-induced AAA model was established, as described previously^23,24^.

The AngII-induced AAA model was established by subcutaneous implantation of an osmotic pump. Briefly, 10-week-old mice were anesthetized with isoflurane, and a small incision was made in the dorsal neck skin to bluntly separate subcutaneous tissue. A mini osmotic pump (model 2004W, 4-week delivery; RWD Life Science, Shenzhen, China) loaded with AngII (1 μg/kg/min) or saline (0.9% NaCl) was inserted, and the wound was closed with a small animal skin stapler. Mortality was recorded, and surviving mice were euthanized at 4 weeks post-implantation. Although the AngII model closely mimics the pathological features of human AAA, it typically results in suprarenal aneurysms. To complement this and to better replicate the classical infrarenal location of human AAAs, we additionally used the CaCl_2_-induced model, specifically targeting the infrarenal aorta.

The calcium chloride (CaCl_2_)-induced AAA model was produced by periarterial application of CaCl_2_, as described previously. In brief, after induction of anesthesia with 1.5% isoflurane, the infrarenal aorta was exposed via midline laparotomy, carefully isolated, and wrapped with 0.5 mol/l CaCl_2_-soaked gauze for 15 min. The abdominal cavity was irrigated with warm sterile saline before closure. The control group received saline treatment only. All mice were euthanized at 4 weeks postoperation for arterial tissue collection and analysis.

The maximal abdominal aortic diameter was quantified under a stereomicroscope, with arterial tissues either snap-frozen in liquid nitrogen for molecular analyses or fixed in 4% paraformaldehyde for histological examination.

β-NMN (500 mg/kg) was administered intraperitoneally every other day, starting 14 days before AngII/CaCl_2_ induction until sacrifice.

AAV serotype 9 with an SM22α promoter expressing mouse *Sirt7*, *Srf-K150R*, and *Srf-K150Q* was constructed and packaged by WZ Biosciences Inc. (Jinan, China). AAV9 or control empty vector (AAV-EV) was delivered via tail vein injection (4 × 12 vg/kg) 3 weeks before AngII infusion.

### Cell culture

Human aortic smooth muscle cells (HASMCs, ACBRI 716) were obtained from Cell Systems (Seattle, WA, USA) and cultured in Complete Classic Medium supplemented with Serum and CultureBoost™ (Cell Systems, 4Z0-500). Cells within passages 3–8 were utilized. Mouse aortic smooth muscle cells (MASMCs, YPC-M019) and rat embryonic thoracic aortic smooth muscle cell line (A7r5, YCL-0020) were acquired from YZFBIO (Shanghai, China). MASMCs were maintained in Smooth Muscle Cell Medium (ScienCell, 1101) containing 10% (v/v) fetal bovine serum (Gibco, A5670701) and 1% (v/v) penicillin–streptomycin (p/s, Gibco, 15070063), with passages 3–6 used experimentally. HEK293T cells (ATCC) and A7r5 cells were cultured in Dulbecco's modified Eagle's medium (Gibco) with identical supplements. A7r5 cells served for stable knockout line generation, whereas HEK293T cells supported viral packaging. All cultures were kept at 37 °C under 5% CO_2_.

### Plasmid construction

For protein overexpression, human genes (*Flag-SIRT7*, *HA-SRF*, and *GST-SRF*) were subcloned into pcDNA3.1. Knockdown constructs targeting the target genes were generated in pLKO.1. Full-length *SIRT7/SRF* genes from multiple species were cloned into cytomegalovirus-driven lentiviral vectors for packaging in HEK293T cells. Key mutants were created using the Mut Express II kit (Vazyme), with all plasmids sequence-verified. Lentiviruses were produced by polyethylenimine-mediated co-transfection (transfer vector: pMD2.G: psPAX2 = 4:3:1), and supernatants were concentrated by ultracentrifugation (see Supplementary Table 4 for details).

### Construction of cell lines

The Srf-knockout lentivirus was produced using the lenti-CRISPR-sgSrf/pMD2.G/psPAX2 three-plasmid system. A7r5 cells were infected with viral particles for 12 h before medium replacement. Puromycin selection (2 μg/ml, 72 h duration) started at 48 h post-infection. Single-cell clones (A7r5^SRF^^*−/−*^) were established by limiting dilution and validated through western blotting for SRF ablation.

### RNA sequencing

Total RNA of aortic samples (*n* = 3 per group) was isolated using TRIzol (9109, Takara). RNA samples underwent high-throughput sequencing (HpaloX, Shenzhen) after library preparation using the Fast RNA-seq Library Prep Kit V2 (ABclonal, RK20306). Sequencing was performed on an Illumina NovaSeq X Plus platform. Differentially expressed genes were identified with DESeq2 (R package) using thresholds of false discovery rate-adjusted P < 0.05 and |log2FC | ≥1. Volcano plots were generated using ggplot2 R package. Heatmap was generated using the pheatmap R package. DAVID tools were used for the Kyoto Encyclopedia Genes and Genomes pathways enrichment analysis (https://david.ncifcrf.gov/).

### Co-immunoprecipitation (Co-IP)

Cells were harvested and lysed with NP-40 lysis buffer (P0013F, Beyotime). After protein quantification, 500 μg of protein was incubated with primary antibodies or IgG rotating overnight at 4 °C. The immunocomplexes were captured using Protein A/G Magnetic beads (HY-K0202, MedChemExpress) for 2 h. Beads were washed four times with ice-cold NP-40 buffer. Protein complexes were eluted with SDS loading buffer for further analysis. The antibodies information is detailed in Supplementary Table 1.

### Chromatin immunoprecipitation (ChIP)

ChIP was performed using ChIP Assay Kit (P2080S, Beyotime), according to the manufacturer’s protocol. Briefly, cells were fixed with 1% formaldehyde to crosslink the target protein and genomic DNA at 37 °C for 10 min, followed by 0.1% glycine to terminate the crosslinking at room temperature for 5 min. Nuclei were isolated and chromatin was sheared by sonication (22% amplitude, 10 s pulse/30 s interval, 16 cycles) to generate 200–1,000 bp fragments. After overnight IP (4 °C) with target antibodies, complexes were captured using ChIP-grade Protein A/G magnetic beads (4 °C, 60 min), eluted with a salt gradient, and digested with proteinase K (45 °C, 1 h). Purified DNA was analyzed by quantitative PCR with gene-specific primers. Putative transcription factor binding sites were predicted via JASPAR (https://jaspar.elixir.no/). The primers used can be obtained from Supplementary Table 4.

### Mass spectrometry

Magnetic bead samples after IP were used to detect the interacting proteins by Q Exactive Plus liquid chromatography tandem mass spectrometry (LC–MS/MS, Wuhan Biobank). Briefly, bead samples were obtained from co-IP and incubated with sodium deoxycholate, Tris (2-carboxyethyl) phosphine, and chloroacetamide at 95 °C for 10 min for protein elution, denaturation, reduction, and alkylation, respectively. The supernatant proteins were digested with trypsin at 37 °C overnight, followed by peptide purification on a styrene-divinylbenzene (SDB) desalting column. Peptide data were collected with Plus LC-MS/MS mass spectrometer and analyzed by MaxQuant software.

### In vitro deacetylation assays

FLAG-SIRT7 was overexpressed in HEK293T cells and purified with Anti-FLAG Affinity Gel (HY-K0217, MedChemExpress). The acetylated SRF-K154ac short peptide was synthesized from ABclonal. For the deacetylation assay, purified SIRT7 protein was incubated with acetylated SRF peptide in deacetylation buffer (50 mM Tris–HCl pH 8.0, 4 mM MgCl_2_, 0.2 mM DTT, 1 mM NAD^+^ and 1× protease inhibitor cocktail) at 37 °C for 1 h. Reaction products were spotted onto polyvinylidene fluoride membranes. The acetylation level of SRF was quantified by dot blot analysis with anti-SRF-K154ac antibodies.

### GST pull-down assay

GST-SRF was overexpressed in HEK293T cells and purified using Glutathione Sepharose 4FF beads (C600911, Sangon Biotech). For GST pull-down assay, purified Flag-SIRT7 was incubated with GST-bound or GST-SRF-bound beads in GST binding buffer (20 mM Tris–HCl, pH 7.4, 0.1 mM ethylenediaminetetraacetic acid, 150 mM NaCl, 0.2% NP-40, protease inhibitor cocktail). Beads were washed, and interacting proteins were eluted with 10 mM reduced glutathione for analysis by Coomassie Brilliant blue staining and immunoblotting.

### Dot blot

To assess antibody efficacy and to conduct in vitro deacetylation experiments, samples were collected and subjected to dot blot analysis. Briefly, equal volumes of the post-reaction samples were spotted onto a polyvinylidene fluoride membrane activated with methanol. The membrane was air-dried at room temperature for 30 min to facilitate protein-membrane crosslinking. After drying, the membrane was blocked with 5% skimmed milk in TBST to prevent nonspecific binding. Subsequently, the membrane was incubated with the primary antibody, followed by washing and incubation with a horseradish peroxidase-conjugated secondary antibody. Signals were visualized using enhanced chemiluminescence. Total protein was assessed by Ponceau S staining according to the manufacturer’s protocol.

### Statistical analysis

All statistical analysis was performed using Prism 9.5 software (GraphPad Software, Inc.). Values were shown as mean ± standard deviation (SD). All statistical data were tested for normality and equality of variance. The unpaired or paired Student’s *t* test was utilized between two groups, and one-way analysis of variance (ANOVA) was used between more than two group comparison followed by Bonferroni test. Multiple groups with two variables were evaluated by two-way ANOVA followed by Bonferroni test. Welch’s correction test was performed for unequal variances, and Mann–Whitney test was applied for variances not normally distributed. Survival analysis was estimated by Kaplan–Meier test. Χ^2^ test or Fisher’s exact test was used for contingency tables. Spearman test was used to assess the correlation between ordinal data and continuous variables. For all statistical methods, *P* < 0.05 was considered significant: *P < 0.05, **P < 0.01, ***P < 0.001.

## Results

### SIRT7 is downregulated in human and murine AAA

**Fig. 1:**
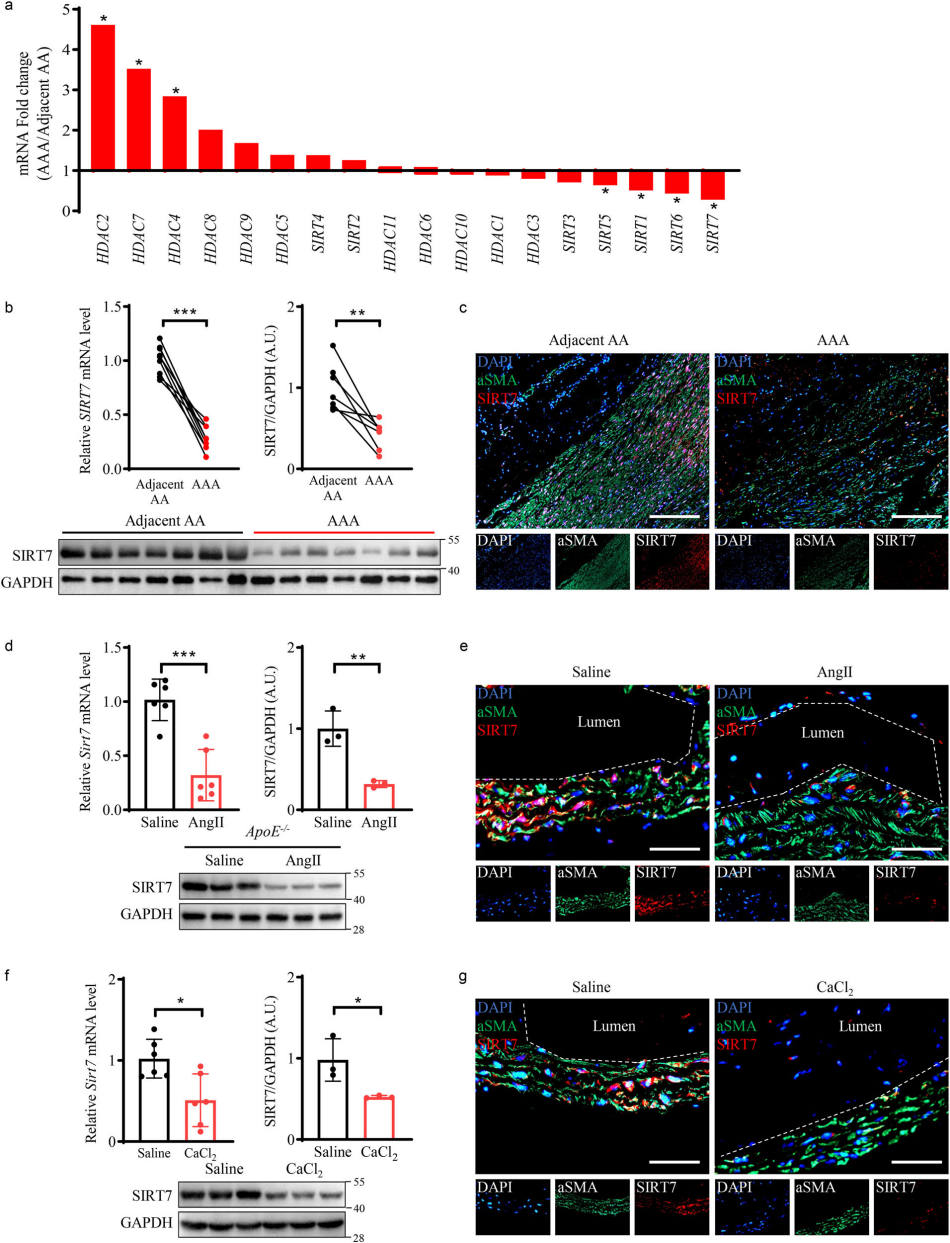
SIRT7 was downregulated in human and murine AAA. **a** Fold change expressions of 18 histone deacetylase (HDAC) isoforms genes measured by real-time-quantitative PCR in human abdominal aortic aneurysm (AAA) and adjacent normal abdominal aorta (adjacent AA). *n* = 9. **b** Relative sirtuin 7 (SIRT7) mRNA and protein expression were determined in human AAA and adjacent AA. *n* = 9 for mRNA and *n* = 7 for protein. **c** Representative immunofluorescence staining images of SIRT7 in human AAA and adjacent AA. Scale bar, 200 μm. **d** Relative Sirt7 mRNA and protein expression were measured in angiotensin II (AngII)-induced AAA model. *n* = 6 for mRNA and *n* = 3 for protein. **e** Representative immunofluorescence staining images of SIRT7 in AngII-induced AAA model. Scale bar, 50 μm. **f** Relative Sirt7 mRNA and protein expression were measured in CaCl_2_-induced AAA model. *n* = 6 for mRNA and *n* = 3 for protein. **g** Representative immunofluorescence staining images of SIRT7 in CaCl_2_-induced AAA model. Scale bar, 50 μm. Data are expressed as mean ± SD. **a**, **b**, Two-tailed paired Student’s *t* test. **d**, **f**, Two-tailed unpaired Student’s *t* test. a.u., arbitrary unit; DAPI, 4′,6-diamidino-2-phenylindole.

To identify HDAC family members involved in AAA pathogenesis, we analyzed the expression of all 18 HDAC isoforms in human AAA lesions and paired adjacent abdominal aorta (adjacent AA) tissues. Among them, SIRT7 exhibited the most pronounced reduction in human AAA (Fig. 1a). Although several other HDACs and sirtuins (for example, SIRT1/5/6, HDAC2/7) also showed significant alterations consistent with previous literature, we focussed on SIRT7 owing to its previously unexplored role in AAA and our promising preliminary functional data. Both mRNA and protein levels of SIRT7 were significantly reduced in aneurysmal tissues (Fig. 1b; patient characteristics are shown in Supplementary Table 2), and immunofluorescence confirmed decreased SIRT7 expression in human AAA specimens (Fig. 1c). Similar downregulation of SIRT7 was observed in two independent murine AAA models, including AngII/*ApoE*^*−/−*^ mice (Fig. 1d, e) and CaCl_2_-induced AAA model (Fig. 1f, g), demonstrating a conserved downregulation of SIRT7 during aneurysm development.

### Global and VSMC-specific SIRT7 deletion promotes AAA formation

The decline of SIRT7 expression in both murine and human AAA samples prompted us to investigate its involvement in AAA formation. We generated global *Sirt7* knockout mice (*Sirt7*^*−/−*^) and established *ApoE*^*−/−*^*Sirt7*^*−/−*^ double-knockout mice with AngII infusion and control *ApoE*^*−/−*^*Sirt7*^*+/+*^ littermates (Supplementary Fig. 1a–c). Global Sirt7 deficiency led to larger aortic diameters, increased AAA incidence, rupture rate, and mortality, accompanied by more severe elastin degradation, without affecting blood pressure, body weight, or lipid profiles (Supplementary Fig. 1d–n). Global Sirt7 deficiency also caused similar aggravation in CaCl_2_-induced AAA mice (Supplementary Fig. 2a–d). Collectively, our findings demonstrate that SIRT7 deficiency is a critical determinant of AAA pathogenesis.

**Fig. 2:**
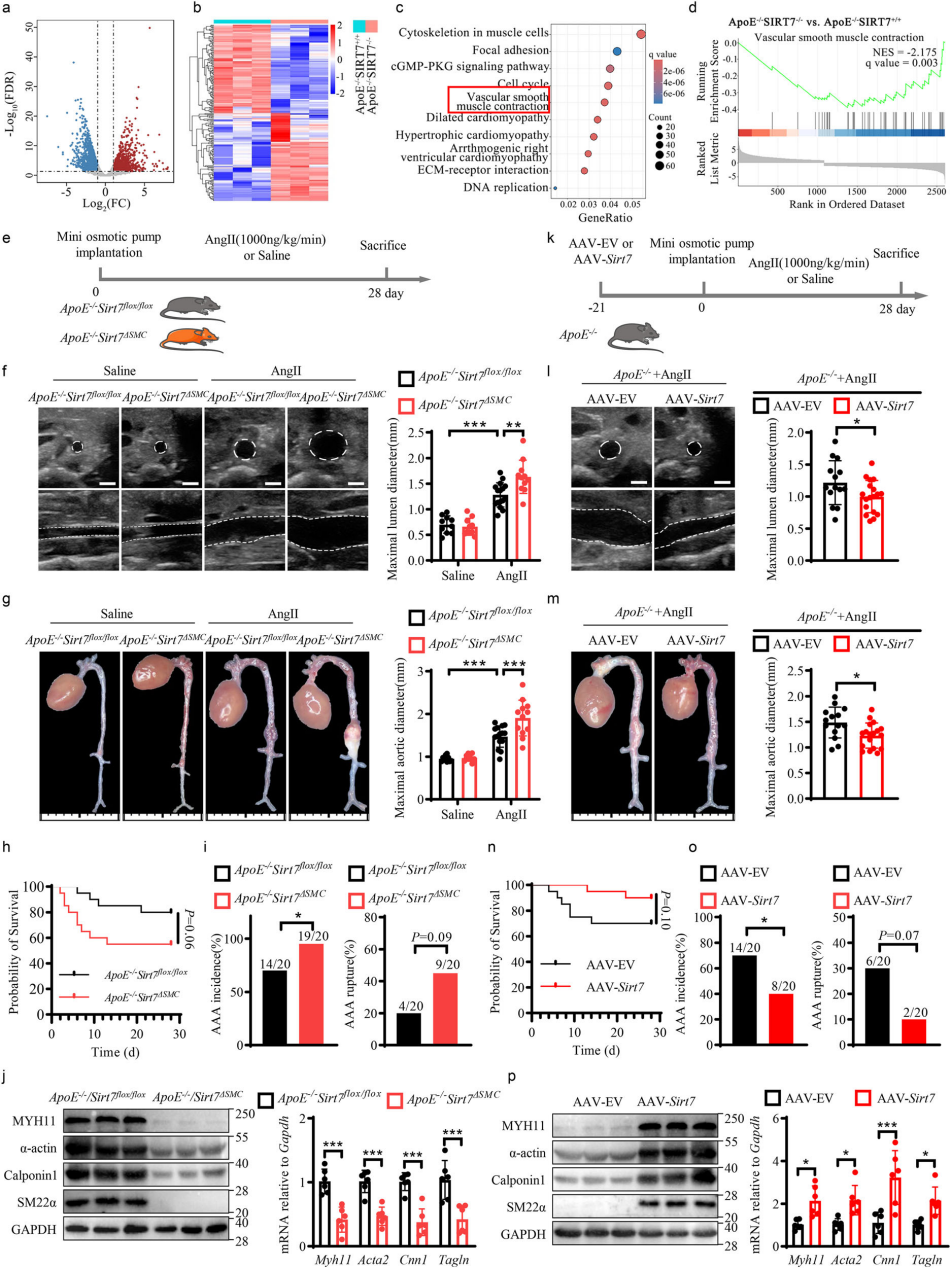
SIRT7 protected against AngII-induced AAA formation via modulating VSMC phenotypic switching. **a**–**d** *ApoE*^*−/−*^*Sirt7*^*+/*+^ and *ApoE*^*−/−*^*Sirt7*^*−/−*^ mice were infused with angiotensin II (AngII) (1,000 ng/kg/min) for 4 weeks. Aneurysmal tissues were collected for RNA sequencing. **a** Volcano plot showing differentiated expressed genes (DEGs). Upregulated genes are shown in red dot (*n* = 966), and downregulated genes are displayed in blue dot (*n* = 1,310). DEGs were defined as genes with a Benjamini–Hochberg adjusted *P*-value < 0.05 and |log_2_(Fold change) |≥1. *n* = 3 per group. **b** Heatmap of DEGs in *ApoE*^*−/−*^*Sirt7*^*+/*+^ and *ApoE*^*−/−*^*Sirt7*^*−/−*^ mice after AngII infusion. *n* = 3 per group. **c** Among the top 10 KEGG pathways of DEGs, vascular smooth muscle contraction (VSMC) was enriched. **d** GSEA of VSMC pathway in *ApoE*^*−/−*^*Sirt7*^*+/*+^ and *ApoE*^*−/−*^*Sirt7*^*−/−*^ mice after AngII infusion. NES, Normalized enrichment score. **e**–**j** *ApoE*^*−/−*^*Sirt7*^*flox/flox*^ and *ApoE*^*−/−*^*Sirt7*^Δ*SMC*^ mice were infused with saline or AngII (1,000 ng/kg/min) for 4 weeks. **e** Schematic diagram of the AngII-induced abdominal aortic aneurysm (AAA) model in *ApoE*^*−/−*^*Sirt7*^*flox/flox*^ and *ApoE*^*−/−*^*Sirt7*^Δ*SMC*^ mice. **f** Representative ultrasonic images and quantitative comparison of maximal lumen diameter in *ApoE*^*−/−*^*Sirt7*^*flox/flox*^ and *ApoE*^*−/−*^*Sirt7*^Δ*SMC*^ mice after 28 days of AngII infusion. *n* = 10 for saline and *n* = 20 for AngII. Scale bar, 1 mm. **g** Representative macroscopic images and quantitative comparison of maximal aortic diameter in *ApoE*^*−/−*^*Sirt7*^*flox/flox*^ and *ApoE*^*−/−*^*Sirt7*^Δ*SMC*^ mice after 28 days of AngII infusion. *n* = 10 for saline and *n* = 20 for AngII. **h** Survival curves of AngII-induced AAA in indicated groups. *n* = 20 per group. **i**, The incidence and rupture rate of AngII-induced AAA in indicated groups. *n* = 20 per group. **j**, Relative protein and mRNA expression of SMC phenotypic switching marker (*Myh11*, *Acta2*, *Cnn1*, and *Tagln*) in *ApoE*^*−/−*^*Sirt7*^*flox/flox*^ and *ApoE*^*−/−*^*Sirt7*^Δ*SMC*^ mice after 28 days of AngII infusion. *n* = 6 for mRNA and *n* = 3 for protein. **k**–**p** *ApoE* mice were injected with AAV-empty vector (EV) or AAV-*Sirt7* via tail vein to overexpress *Sirt7*. Three weeks later, mice were infused with AngII (1,000 ng/kg/min) for 4 weeks to induce AAA model. **k** Schematic diagram of the AngII-induced AAA model in *ApoE*^*−/−*^ mice treated with AAV-EV or AAV-*Sirt7*. **l** Representative ultrasonic images and quantitative comparison of maximal lumen diameter in indicated groups. *n* = 14 for AAV-EV and *n* = 18 for AAV-*Sirt7*. Scale bar, 1 mm. **m** Representative macroscopic images and quantitative comparison of maximal aortic diameter in indicated groups. *n* = 14 for AAV-EV and *n* = 18 for AAV-*Sirt7*. **n** Survival curves of AngII-induced AAA in indicated groups. *n* = 20 per group. **o** The incidence and rupture rate of AngII-induced AAA in indicated groups. *n* = 20 per group. **p** Relative protein and mRNA expression of SMC contractile marker (*Myh11*, *Acta2*, *Cnn1*, and *Tagln*) in *ApoE*^*−/−*^ mice treated with AAV-EV or AAV-*Sirt7* after AngII infusion. *n* = 6 for mRNA and *n* = 3 for protein. Data are expressed as mean ± SD. **f**, **g**, **j**, **p** Two-way analysis of variance followed by Bonferroni multiple comparisons. **h**, **n** Kaplan–Meier method followed by log-rank tests. **i**, **o** Fisher exact test. **l**, **m** Two-tailed unpaired Student’s *t* test. FC, fold change; FDR, false discovery rate; GSEA, Gene Set Enrichment Analysis; KEGG, Kyoto Encyclopedia Genes and Genomes; SIRT7, sirtuin 7.

To understand the underlying mechanisms between SIRT7 and AAA, RNA sequencing of aneurysmal tissues was performed. Results revealed that SIRT7 loss altered gene expression related to VSMC contractility, suggesting that SIRT7 may regulate VSMC phenotype during AAA formation (Fig. 2a–d). Therefore, we generated and validated the VSMC-specific Sirt7 knockout mice (*ApoE*^*−/−*^*Sirt7*^Δ*SMC*^) (Supplementary Fig. 3a–d), and no differences in systemic parameters were observed (Supplementary Fig. 3e–g). VSMC-specific Sirt7 deletion led to increased aortic diameters, AAA incidence, rupture rate, mortality, greater elastin degradation, and loss of contractile markers compared with littermate controls (Fig. 2e–j and Supplementary Fig. 3h). Consistently, *Sirt7*^Δ*SMC*^ also facilitated AAA formation in the CaCl_2_-induced model (Supplementary Fig. 4a–d).

### VSMC-specific SIRT7 overexpression ameliorates AAA formation

To further validate the pathological role of SIRT7 in AAA formation, AAV-mediated SIRT7 (AAV-*Sirt7*) overexpression in *ApoE*^*−/−*^ mice was performed and validated (Supplementary Fig. 5a). Compared with control group mice, SIRT7 overexpression markedly reduced aortic diameters, AAA incidence, rupture rate, mortality, elastin breakdown, and loss of contractile markers (Fig. 2k–p and Supplementary Fig. 5b), without altering systemic parameters (Supplementary Fig. 5c–e). Collectively, these results demonstrate that VSMC-derived SIRT7 acts as a crucial protective factor against aneurysm formation by preserving the VSMC contractile phenotype.

### SIRT7 maintains VSMC contractile phenotype through its deacetylase activity

**Fig. 3:**
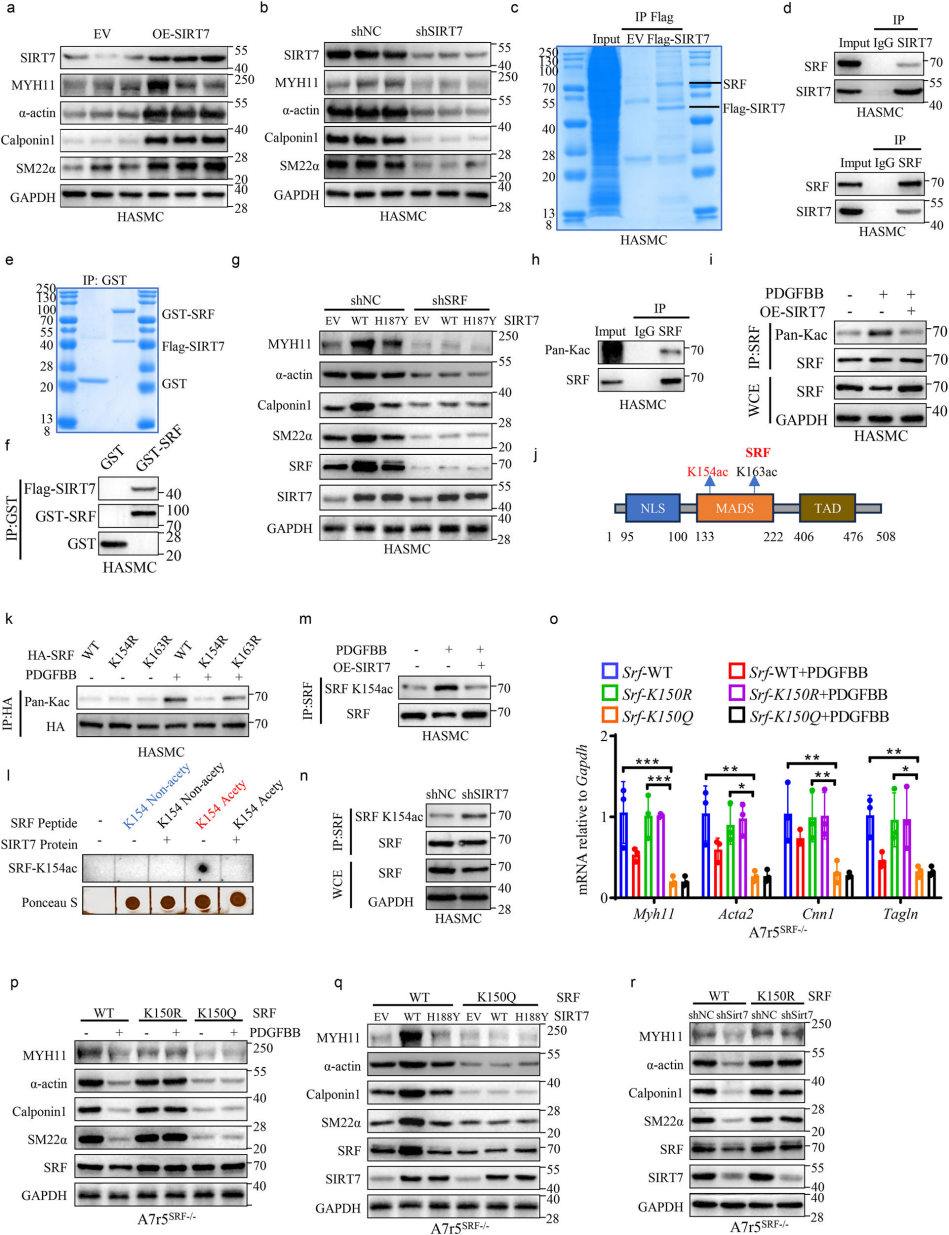
SIRT7 regulated VSMC phenotype switching by deacetylating SRF in vitro. **a** Human aortic smooth muscle cell (HASMC) was infected with lentivirus containing empty vector (EV) or Flag-sirtuin 7 (SIRT7) for 48 h, followed by platelet-derived growth factor (PDGF)-BB treatment for 24 h. The protein expression of SIRT7, MYH11, α-actin, Calponin1, and SM22α was determined by western blotting (WB). *n* = 3. **b** HASMC was infected with lentivirus containing shNC or shSIRT7 for 48 h, followed by serum-free culture for 24 h. The protein expression of SIRT7, MYH11, α-actin, Calponin1, and SM22α was determined by WB. *n* = 3. **c** HASMC was infected with lentivirus containing EV or Flag-SIRT7. Then whole-cell extract (WCE) was collected for immunoprecipitation (IP) with anti-Flag antibody magnetic beads, followed by Coomassie blue staining. **d** Co-IP of endogenous SIRT7 and serum response factor (SRF) in HASMC. **e**, **f** Flag-SIRT7 protein was incubated with GST-SRF or GST protein for the in vitro binding assay. IP was performed with anti-GST antibody magnetic beads, followed by Coomassie blue staining and WB. **g** HASMC was infected with lentivirus overexpressing EV, SIRT7-WT, SIRT7-H187Y inactive mutant. SRFs were silenced with shNC or shSRF for 48 h. HASMC cells were treated with PDGF-BB for 24 h, and WCE was collected for WB analysis. **h** HASMC cell lysates were collected for IP with SRF antibody, followed by WB analysis using Pan-Kac antibody. **i** HASMC was infected with lentivirus containing EV or SIRT7 for 48 h, followed by PDGF-BB treatment for 24 h. WCE was collected for IP with anti-SRF antibody, followed by WB. **j** Mass spectrometry analysis of SRF potential acetylation sites. **k** HASMC was infected with HA-SRF wild-type (WT) or mutant plasmids as indicated for 48 h, followed by PDGF-BB treatment for 24 h. WCE was then collected for IP with anti-HA antibody magnetic beads, followed by WB. **l** To conduct in vitro deacetylation assays, Flag-SIRT7 proteins were purified as the source of deacetylase and the synthetic SRF-K154 acetylation peptides as the substrate. SRF-K154 non-acetylation peptides (blue) as a negative control and SRF-K154 acetylation peptides (red) as a positive control. **m**, **n** HASMC was infected with lentivirus containing EV, OE-SIRT7 or shNC, shSIRT7 for 48 h, followed by PDGF-BB treatment or serum starvation for 24 h. WCE was collected for IP with anti-SRF antibody, followed by WB. **o**, **p** A7r5^SRF^^−/−^ was transfected with SRF-WT or mutant plasmids as indicated. After 48 h, the cells were treated with or without PDGF-BB for 24 h. mRNA and protein expression of *Myh11*, *Acta2*, *Cnn1*, and *Tagln* were determined by qPCR and WB, respectively. **q** A7r5^SRF^^−/−^ was transfected with EV, SIRT7-WT, or H188Y inactive mutant plasmids and SRF was reconstructed by SRF-WT or SRF-K150Q. After 48 h, the cells were treated with PDGF-BB for 24 h. WCEs were collected for WB analysis. **r** Sirt7 in A7r5^SRF^^−/−^ was silenced with shNC or shSirt7 and SRF was reconstructed with SRF-WT or SRF-K150R for 48 h. Cells were serum-starved for 24 h and collected for WB analysis. All immunoblotting was independently performed three times. Data are expressed as mean ± SD. **o** One-way analysis of variance followed by Bonferroni post hoc test. MADS, MCM1, Agamous, Deficiens, and Srf DNA-binding domain; NLS, nuclear localization site; TAD, transcriptional activation domain.

We established a phenotypic switching cell model in vitro to clarify the regulatory role of SIRT7 on the VSMC differentiation. *Sirt7* and contractile genes were downregulated in platelet-derived growth factor (PDGF)-BB-induced synthetic VSMCs and upregulated in serum starvation-induced contractile VSMCs^25^ (Supplementary Fig. 6a, b). SIRT7 overexpression increased, whereas SIRT7 knockdown suppressed, contractile gene expression in HASMCs (Fig. 3a, b and Supplementary Fig. 6c–f), with similar results in MASMCs (Supplementary Fig. 6g, h).

Given SIRT7’s NAD^+^-dependent deacetylase activity^26^, we tested whether its enzymatic function was required for this regulation. Overexpression of SIRT7-WT, but not the catalytically inactive SIRT7-H187Y (*Homo sapiens*)/H188Y (*Mus musculus*) mutants^27^, restored contractile marker expression in PDGF-BB-treated VSMCs (Supplementary Fig. 7a, d), indicating that the deacetylase activity of SIRT7 is essential.

### SIRT7 directly deacetylates SRF-K154 to maintain VSMC contractile phenotype

To identify substrates of SIRT7 deacetylase^28^, we performed co-IP and MS in HASMC, which revealed an interaction between SIRT7 and SRF, the master transcription factor governing VSMC contractile genes (Supplementary Data 1, Fig. 3c, d, and Supplementary Fig. 8a). Data in HEK293T and MASMC also suggested that SIRT7 could bind with SRF (Supplementary Fig. 8b, c). An in vitro GST pull-down assay further demonstrated direct binding between SIRT7 and SRF (Fig. 3e, f). Indeed, promotive effect of SIRT7 on maintaining VSMC contractile phenotype was abolished by SRF knockdown (Fig. 3g and Supplementary Fig. 8d), further confirming that SIRT7 interacts with SRF to maintain VSMC contractility. Therefore, we concluded that SRF is the potential substrate of SIRT7 deacetylase when modulating VSMC phenotypic switching.

Given that SIRT7 is a deacetylase, we hypothesized that SRF may undergo SIRT7-mediated deacetylation to modulate VSMC phenotypic switching^29^. To determine whether SRF undergoes acetylation, we performed co-IP assays to isolate SRF from HASMCs and detect its acetylation using a pan-lysine acetylation antibody (Pan-Kac). Notably, SRF acetylation was detected in HASMCs and increased upon PDGF-BB stimulation, but was reversed by SIRT7 overexpression (Fig. 3h, i and Supplementary Fig. 8e). Furthermore, as other sirtuin family members such as SIRT1 and SIRT6 are also known to exert protective effects against AAA, we sought to determine whether the deacetylation of SRF is specific to SIRT7. Results revealed that SRF acetylation was exclusively increased by SIRT7 knockdown, whereas silencing of SIRT1 or SIRT6 had no significant effect (Supplementary Fig. 8f). This indicates that SIRT7 specifically deacetylates SRF. MS was performed to screen the potential acetylation sites on SRF, and two sites, K154 and K163, were identified. Data suggested that only the K154R mutant but not K163R could abolish PDGF-BB-induced SRF acetylation (Supplementary Data 2, Fig. 3j, k, and Supplementary Fig. 8g). Given that the K154 (*H. sapiens)*/K150 (*M. musculus*) site is highly conserved across species (Supplementary Fig. 8h), we hypothesized that SRF-K154 is the catalytic substrate of SIRT7. Next, K154-specific acetylation antibody (anti-SRF-K154ac) was generated (Supplementary Fig. 8i). In vitro deacetylation assays confirmed that SIRT7 directly deacetylated SRF at K154 (Fig. 3l). Consistently, SIRT7 overexpression decreased, whereas SIRT7 silencing enhanced, the SRF-K154 acetylation in HASMCs (Fig. 3m, n).

### SRF-K154 deacetylation facilitates maintaining VSMC contractile phenotype

To elucidate the role of SRF-K154 acetylation (K150 in mice) in VSMC phenotypic switching, we generated SRF-deficient A7r5 cells (A7r5^SRF−/−^) and reintroduced WT, deacetylation-mimetic (K150R), or acetylation-mimetic (K150Q) SRF mutants (Supplementary Fig. 8j, k). PDGF-BB treatment suppressed VSMC contractile markers in SRF-WT cells, whereas SRF-K150R restored their expression even under PDGF-BB stimulation. By contrast, SRF-K150Q failed to induce contractile gene expression (Fig. 3o, p and Supplementary Fig. 8l). Therefore, we hypothesized that SIRT7 regulates VSMC phenotypic switching in an SRF-K154 deacetylation-dependent manner. Moreover, in *A7r5*^*SRF−/−*^ cells, SRF-K150Q abrogated, while SRF-K150R enhanced, the promotive effect of SIRT7 on contractile gene expression even when SIRT7 was silenced (Fig. 3q, r and Supplementary Fig. 8m, n). Collectively, these data demonstrate that SIRT7 preserves the VSMC contractile phenotype by deacetylating SRF at K154.

### SIRT7 deacetylates and stabilizes SRF by inhibiting ubiquitination-mediated degradation

**Fig. 4:**
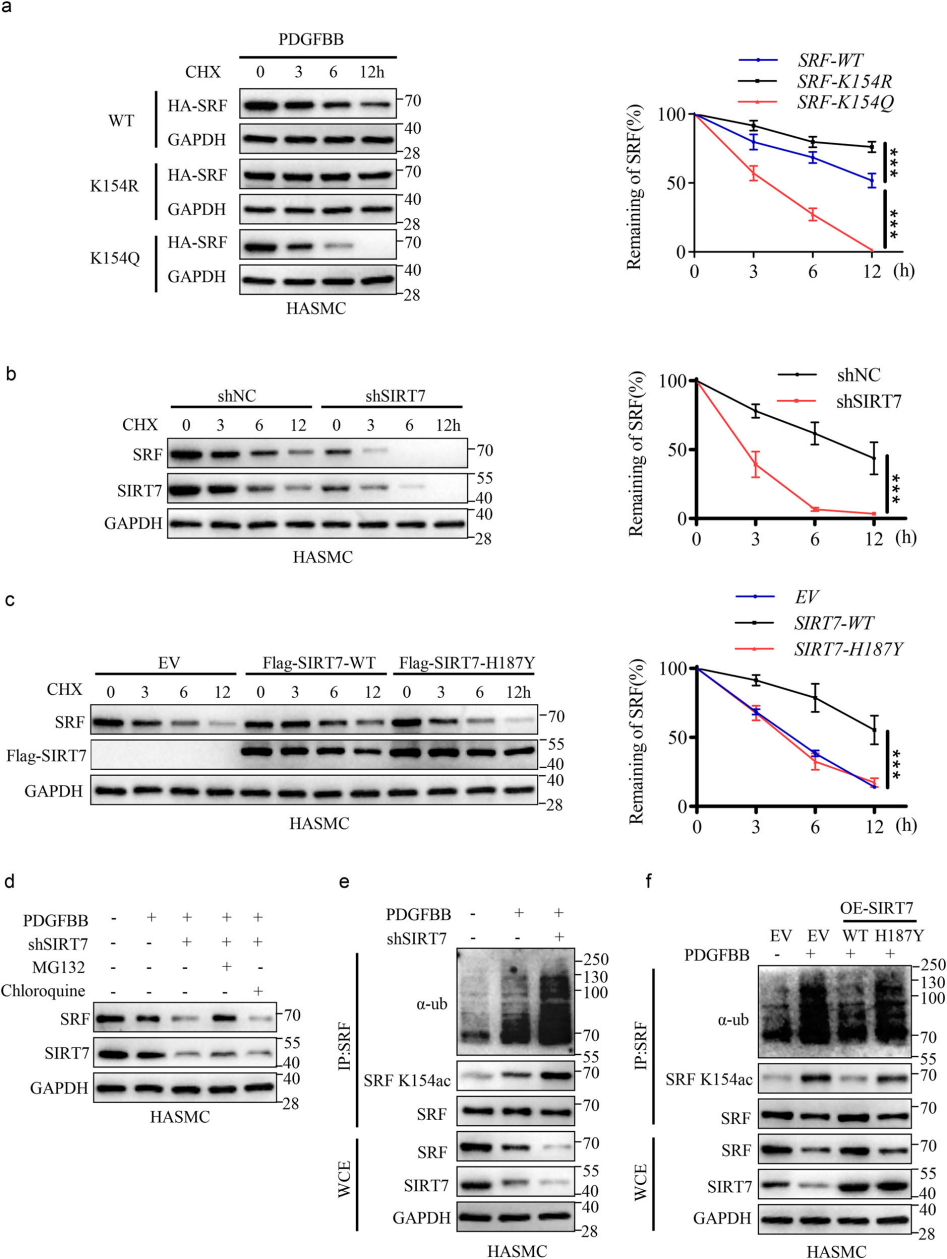
SIRT7 deacetylates and stabilizes SRF by inhibiting ubiquitination-mediated degradation. **a** Human aortic smooth muscle cell (HASMC) was infected with lentivirus containing serum response factor (SRF)-wild-type (WT), SRF-K154R, or SRF-K154Q for 48 h, followed by platelet-derived growth factor (PDGF)-BB treatment for 24 h. Then the cells treated with cycloheximide (CHX, 10 μg/ml) for 0, 3, 6, and 12 h. Whole-cell extract (WCE) was collected for western blot (WB) analysis of SRF, and percentage of SRF remained at each time point was calculated. *n* = 3. HASMC was infected with lentivirus containing shNC, shSIRT7 (part **b**) or empty vector (EV), SIRT7-WT, SIRT7-H187Y inactive mutant (part **c**) for 48 h, followed by serum starvation or PDGF-BB treatment for 24 h. Then cells were treated with CHX (10 μg/ml) for 0, 3, 6, and 12 h. WCE was collected for WB analysis of SRF, and percentage of SRF remained at each timepoint was calculated. *n* = 3. **d** HASMC was infected with lentivirus containing shNC or shSIRT7. The cells were pretreated with MG132 (proteasome inhibitor, 10 μM) or chloroquine (lysosome inhibitor, 30 μM) for 2 h, followed by PDGF-BB treatment for 24 h. WCE was collected for WB analysis. *n* = 3. HASMC was infected with lentivirus containing shNC, shSIRT7 (part **e**) or EV, SIRT7-WT, SIRT7-H187Y inactive mutant (part **f**) for 48 h, followed by PDGF-BB treatment for 24 h. WCE was collected for immunoprecipitation (IP) with SRF antibody followed by WB analysis. *n* = 3. Data are expressed as mean ± SD. **a**–**c** Two-way analysis of variance followed by Bonferroni post hoc test. SIRT7, sirtuin 7.

Post-translational modifications of non-histone proteins have critical roles in regulating protein stability, activity, subcellular localization, and protein–protein interactions^30^. Given that SIRT7 modulates SRF-K154 acetylation levels, we wondered whether SIRT7-mediated SRF-K154 acetylation affects its stability. Cycloheximide (CHX)-chase assays revealed accelerated SRF degradation in cells expressing acetylation-mimetic *SRF*-K154Q, whereas decreased SRF degradation in cells expressing deacetylation-mimetic *SRF*-K154R (Fig. 4a). Furthermore, SIRT7 knockdown promoted SRF degradation, whereas *SIRT7*-WT, but not its deacetylase-deficient mutant H187Y, increased SRF accumulation in both HASMCs (Fig. 4b, c) and MASMCs (Supplementary Fig. 9a, b). These data suggest that SIRT7-mediated SRF deacetylation inhibits SRF degradation.

Protein degradation is achieved primarily through two pathways: the ubiquitin-dependent proteasome pathway and the autophagy-dependent lysosomal pathway^31^. Treatment with the proteasome inhibitor MG132, but not the lysosomal inhibitor chloroquine, restored SRF levels under SIRT7 deficiency; this indicated that a ubiquitin–proteasome-dependent degradation pathway may be involved in SRF degradation (Fig. 4d and Supplementary Fig. 9c). Moreover, PDGF-BB-induced SRF acetylation and ubiquitination were enhanced by SIRT7 knockdown while reversed by *SIRT7*-WT overexpression both in HASMCs (Fig. 4e, f and Supplementary Fig. 9d, e) and in MASMCs (Supplementary Fig. 9f, g).

### SIRT7 suppresses XPO1-mediated SRF nucleus-to-cytoplasmic translocation

**Fig. 5:**
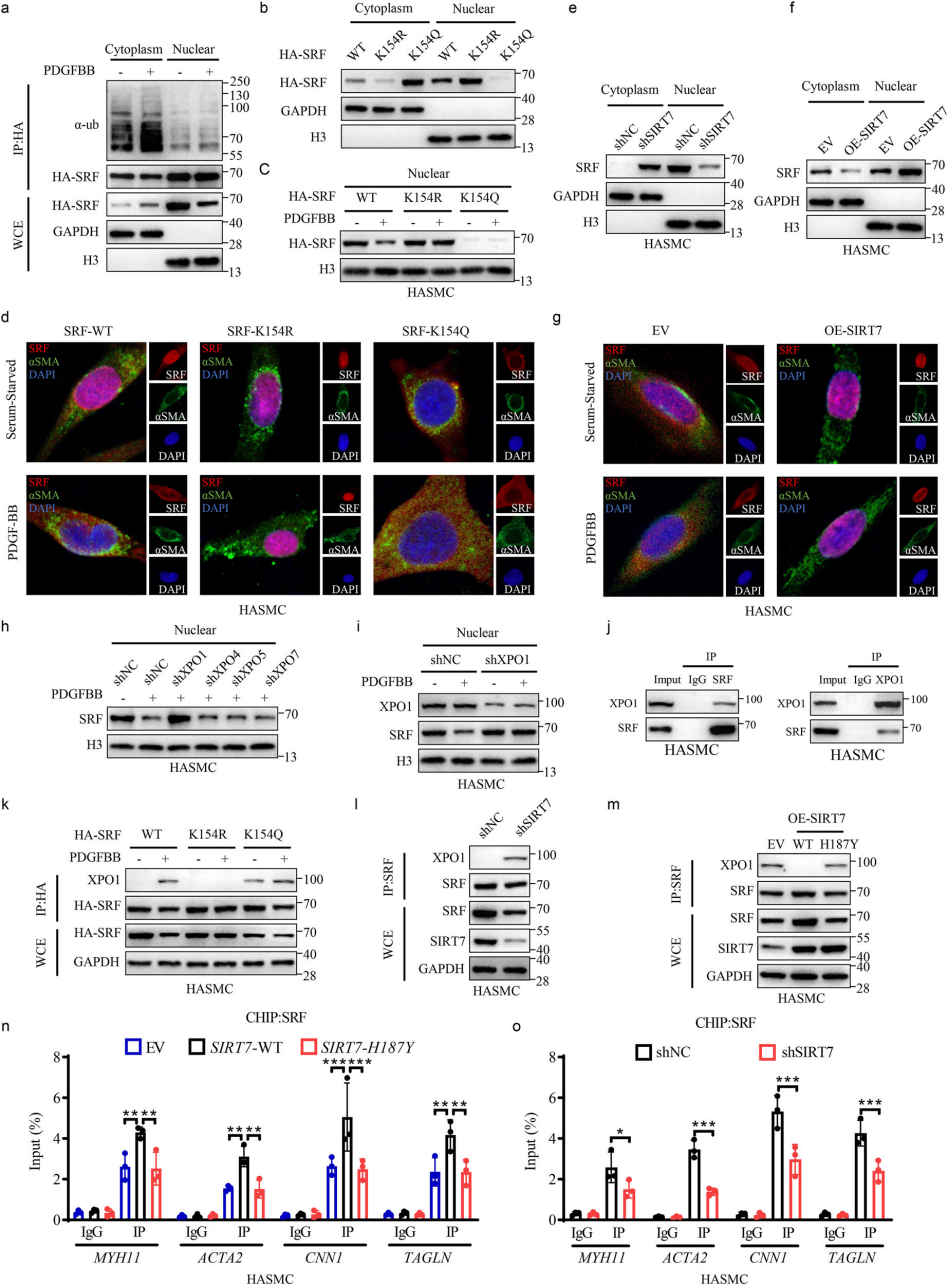
SIRT7 suppresses XPO1-mediated SRF nucleus-to-cytoplasmic translocation. **a** Human aortic smooth muscle cell (HASMC) was infected with lentivirus containing HA-serum response factor (SRF) for 48 h, followed by platelet-derived growth factor (PDGF)-BB treatment for 24 h. Cytosolic and nuclear fractions were collected for immunoprecipitation (IP) with HA antibody, followed by western blot (WB) analysis. **b** Cytosolic and nuclear fractions from HASMC overexpressing HA-SRF wild-type (WT) or mutants were collected and subjected to WB analysis. **c** HASMCs were infected with HA-SRF WT or mutants and were treated with PDGF-BB. Nuclear fractions were collected and subjected to WB analysis. **d** HASMCs were infected with lentivirus containing HA-SRF WT or HA-SRF-K154R/Q mutants for 48 h, followed by serum starvation or PDGF-BB treatment for 24 h. Immunofluorescence staining images showed the cellular localization of SRF. **e**, **f** Sirtuin 7 (SIRT7) in HASMC was silenced with shNC or shSIRT7 and was overexpressed with empty vector (EV) or SIRT7. Cytosolic and nuclear fractions were collected and subjected to WB analysis. **g** HASMC was infected with lentivirus containing EV or SIRT7 for 48 h, followed by serum starvation or PDGF-BB treatment for 24 h. Immunofluorescence images show the cellular localization of SRF. **h** XPO1/4/5/7 in HASMC was silenced with shXPO1, shXPO4, shXPO5, or shXPO7, respectively. HASMCs were treated with PDGF-BB; nuclear fractions were collected and subjected to WB analysis. **i** HASMC was silenced with shXPO1 and was treated with PDGF-BB. Nuclear fractions were collected and subjected to WB analysis. **j** Co-IP of endogenous XPO1 and SRF in HASMC. **k** HASMC was infected with lentivirus containing HA-SRF WT or HA-SRF-K154R/Q mutants for 48 h, followed by serum starvation or PDGF-BB treatment for 24 h. Whole-cell extract (WCE) was collected for IP with HA antibody, followed by WB analysis. HASMC was infected with lentivirus containing shNC, shSIRT7 (part **l**), EV, SIRT7 WT, or SIRT7-H187Y inactive mutant (part **m**). WCE was collected for IP with SRF antibody, followed by WB analysis. **n**, **o** HASMC was infected with lentivirus containing shNC, shSIRT7, EV, SIRT7-WT, or SIRT7-H187Y for 48 h, followed by serum starvation or PDGF-BB treatment for 24 h. The cell extracts were collected for chromatin IP with anti-SRF antibody, followed by qPCR of MYH11, ACTA2, CNN1, and TAGLN gene promoters. *n* = 3. All immunoblotting was independently performed three times. Two-way analysis of variance followed by Bonferroni post hoc test. DAPI, 4′,6-diamidino-2-phenylindole.

Given that ubiquitin-dependent protein degradation occurs both in cytoplasm and in nucleus^32,33^, we performed experiments to determine the subcellular location of SRF degradation. Data showed that SRF ubiquitination mainly occurred in the cytoplasm and increased upon PDGF-BB stimulation (Fig. 5a and Supplementary Fig. 10a). Since the transcription factor SRF is predominantly localized to the nucleus^34^, we hypothesized that SRF acetylation/deacetylation levels modulate nucleus–cytoplasmic translocation. Indeed, SRF-K154R localized predominantly in the nucleus, whereas SRF-K154Q mainly accumulated in the cytoplasm in both HASMCs (Fig. 5b–d and Supplementary Fig. 10b, c) and MASMCs (Supplementary Fig. 10d–g). PDGF-BB-induced nuclear export of SRF was enhanced by SIRT7 knockdown, whereas it is reversed by SIRT7 overexpression (Fig. 5e–g and Supplementary Fig. 10h, i). These data suggest that SIRT7 suppresses SRF nucleus-to-cytoplasmic translocation.

Large nuclear proteins such as SRF depend on exportins such as exportin 1/4/5/7 (XPO1/4/5/7) to transport across the nuclear membrane^35–37^. As shown in Fig. 5h–j, in HASMCs, only XPO1 knockdown blocked PDGF-BB-induced SRF nuclear export (Supplementary Fig. 10l, m). Similar results were also remained in MASMCs (Supplementary Fig. 10j, k). Experiments in HASMCs showed that SRF binding to XPO1 was strengthened by SRF-K154 acetylation, SIRT7 deficiency, or SIRT7 inactive mutant H87Y; however, SRF-XPO1 binding was weakened by SRF-K154 deacetylation or SIRT7 overexpression (Fig. 5k–m). Similar results were obtained in MASMC experiments (Supplementary Fig. 10n). These findings indicate that SIRT7-mediated SRF-K154 deacetylation inhibits SRF nucleus-to-cytoplasmic translocation and following ubiquitin-mediated degradation.

Since the K154 acetylation site lies within the MADS DNA-binding domain (Fig. 3j), a functional region essential for transcription factor binding to genomic DNA, we assessed whether SRF transcriptional activity could be affected by acetylation/deacetylation. Chromatin immunoprecipitation showed that SIRT7-WT, but not its inactive mutant H187Y, increased SRF binding to the promoters of contractile genes (Myh11, Acta2, Cnn1, and Tagln), whereas SIRT7 silencing reduced this binding (Fig. 5n, o and Supplementary Fig. 11a, b). These results demonstrated that SRF-K154 deacetylation by SIRT7 enhanced its transcriptional activity.

### Overexpression of deacetylated SRF moderates AAA formation

**Fig. 6:**
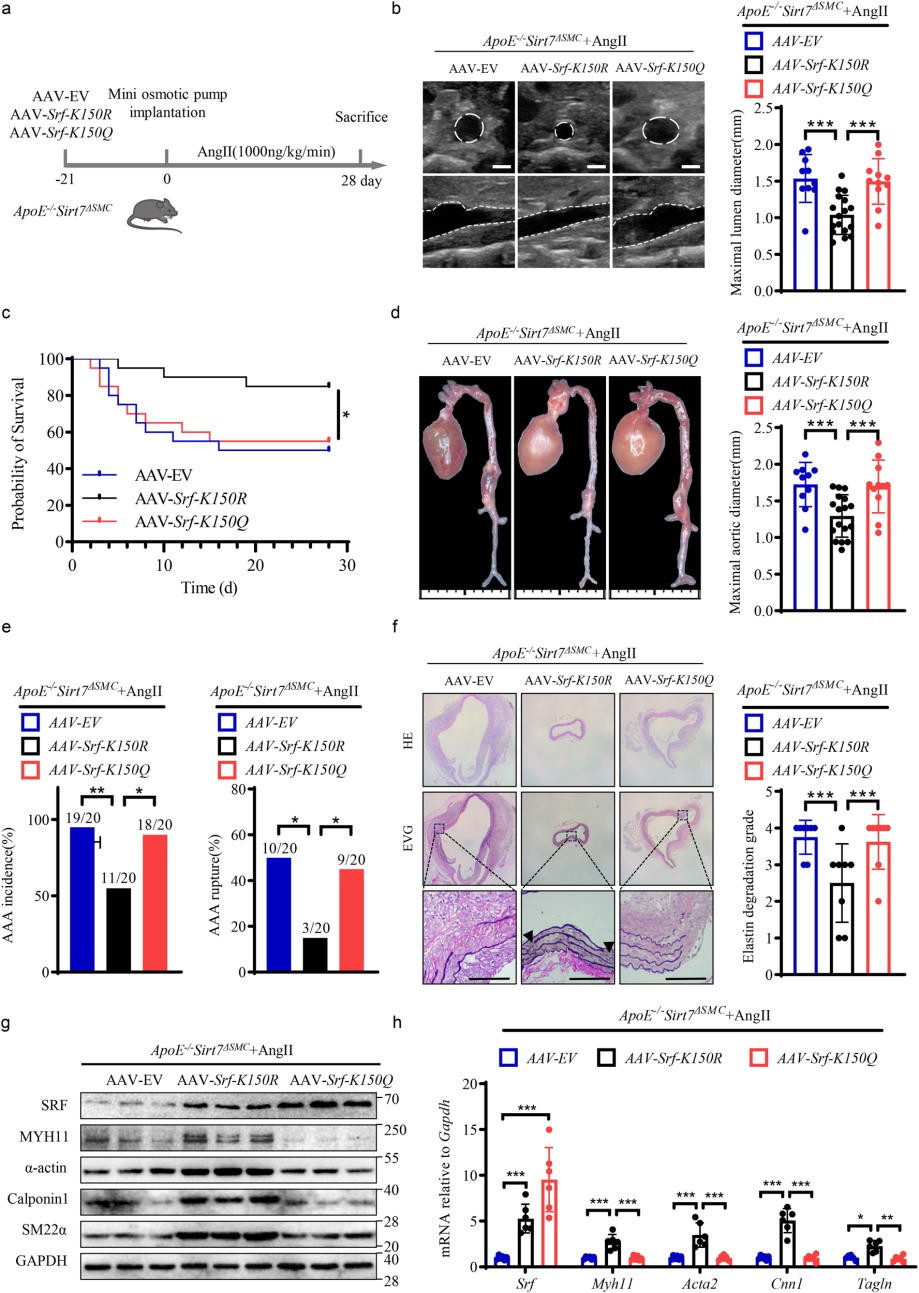
Overexpression of deacetylated SRF-K154 moderates aortic aneurysm in AngII-infused *ApoE*^*−/−*^*Sirt7*^Δ*SMC*^ mice. **a** Schematic diagram of the angiotensin II (AngII)-induced abdominal aortic aneurysm (AAA) model in *ApoE*^*−/−*^*Sirt7*^Δ*SMC*^ mice after administration of AAV-empty vector (EV), AAV-*Srf-K150R*, or AAV-*Srf-K150Q*. **b** Representative ultrasonic images and quantitative comparison of maximal lumen diameter in AngII-induced AAA. *n* = 20 per group. Scale bar, 1 mm. **c** Survival curves and the rupture rate of AngII-induced AAA in indicated groups. *n* = 20 per group. **d** Representative macroscopic images and quantitative comparison of maximal aortic diameter in AngII-induced AAA. *n* = 20 per group. **e** The incidence and rupture rate of AngII-induced AAA in indicated groups. *n* = 20 per group. **f** Representative HE and VVG staining images and quantitative comparison of VVG staining score in AngII-induced mice. *n* = 8. Scale bar, 50 μm. **g**, **h** mRNA and protein expression of *Srf*, *Myh11*, *Acta2*, *Cnn1*, and *Tagln* were determined in indicated mice. *n* = 6 for RNA and *n* = 3 for protein. Data are expressed as mean ± SD. **b**, **d**, **f** One-way analysis of variance (ANOVA) followed by Bonferroni post hoc test. **c**, Kaplan–Meier method followed by log-rank tests. **e** Fisher exact test. **h** Two-way ANOVA followed by Bonferroni post hoc test. HE, hematoxylin and eosin; SIRT7, sirtuin 7.

To further confirm pathological effect of SRF acetylation in vivo, AAVs expressing *Srf* mutants were introduced into *ApoE*^*−/−*^*Sirt7*^Δ*SMC*^ mice subjected to AngII-induced AAA. Introduction of *Srf* mutants did not alter systemic parameters (Supplementary Fig. 12a–c). *Srf*-K150R, but not *Srf*-K150Q, markedly reduced aortic dilation, AAA incidence, rupture, and mortality, accompanied by preserved elastin integrity and increased contractile marker expression (Fig. 6a, h). These results indicate that SIRT7-mediated SRF-K154 deacetylation prevents AAA progression.

### NMN supplementation protects against AAA formation

**Fig. 7:**
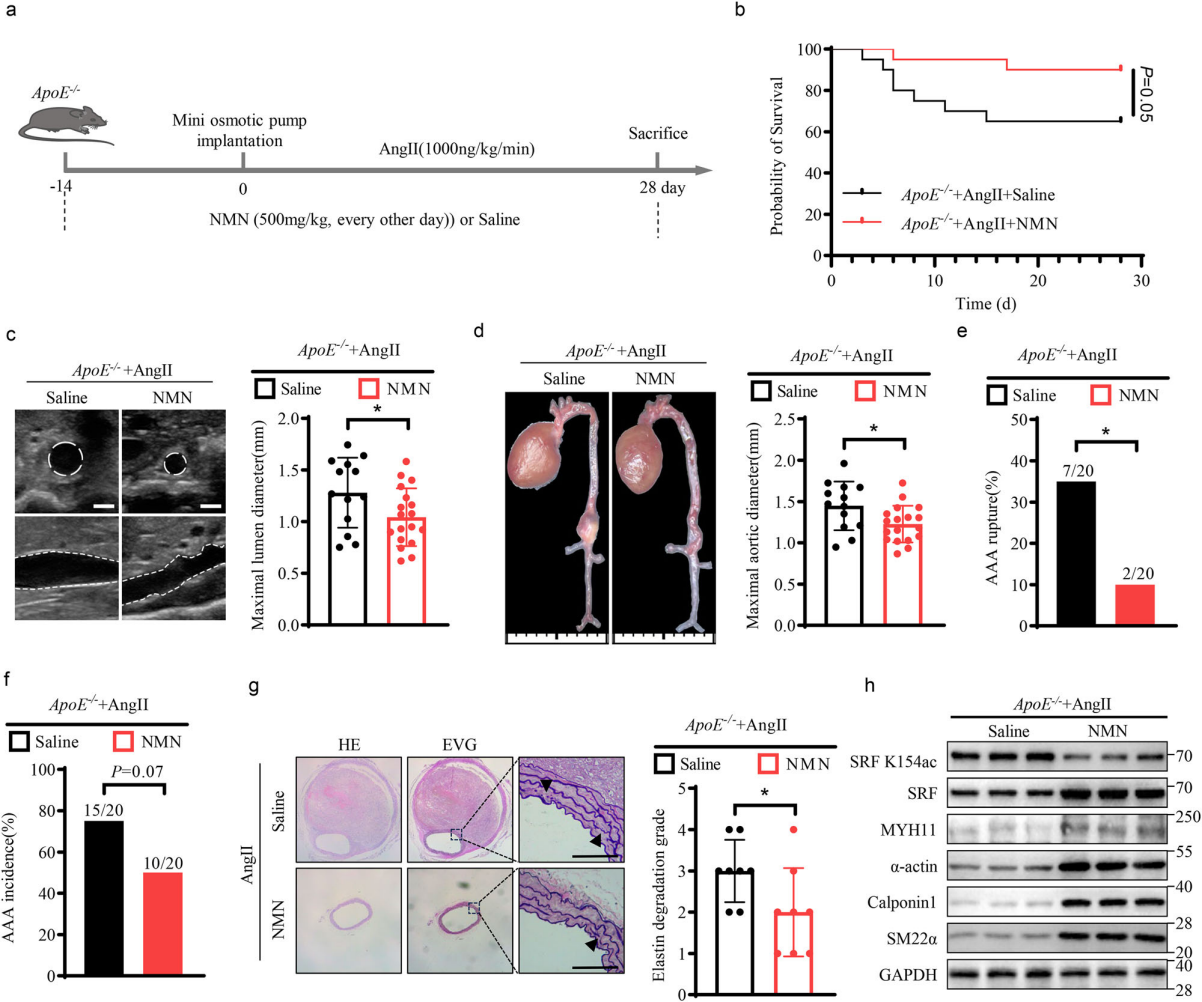
SIRT7 activation by NMN supplementation protects against aortic aneurysm formation in mice. **a** Schematic diagram of the angiotensin II (AngII)-induced abdominal aortic aneurysm (AAA) model in *ApoE*^*−/−*^ mice administrated with saline or nicotinamide mononucleotide (NMN). **b** Survival curves of AngII-induced AAA in indicated groups. *n* = 20 per group. **c** Representative ultrasonic images and quantitative comparison of maximal lumen diameter in AngII-induced mice after NMN administration. *n* = 13 for saline and *n* = 18 for NMN. Scale bar, 1 mm. **d** Representative macroscopic images and quantitative comparison of maximal aortic diameter in AngII-induced mice after NMN administration. *n* = 13 for saline and *n* = 18 for NMN. **e** The rupture rate of AngII-induced AAA in indicated groups. *n* = 20 per group. **f** The incidence of AngII-induced AAA in indicated groups. *n* = 20 per group. **g** Representative hematoxylin and eosin (HE) and VVG staining images and quantitative comparison of VVG staining score in AngII-induced mice after NMN administration. *n* = 8. Scale bar, 100 μm. **h** Aortic tissues from AngII-induced mice after NMN administration were collected for western blotting analysis. *n* = 3. Data are expressed as mean ± SD. **b** Kaplan–Meier method followed by log-rank tests. **c**, **d**, **g** Two-tailed paired Student’s *t* test. **e**, **f** Fisher exact test. SIRT7, sirtuin 7.

Given that SIRT7-mediated SRF-K154 deacetylation maintains VSMC contractility and inhibits AAA progression, we evaluated whether SIRT7 activation could prevent AAA. NMN, an NAD⁺ precursor known to activate SIRT7 (ref. ^38^), was introduced in the AngII-induced *ApoE*^*−/−*^ AAA model (Fig. 7a). As shown in Fig. 7, NMN administration markedly reduced aortic dilation, AAA incidence, mortality, and elastin degradation (Fig. 7b–g) without altering systemic parameters (Supplementary Fig. 13a–c). NMN also restored contractile marker expression (Fig. 7h). Similar protective effect of NMN against AAA was obtained in the CaCl_2_-induced AAA model (Supplementary Fig. 14a–e). In vitro HASMCs tests also revealed that NMN could reverse PDGF-BB-induced loss of contractile markers and increased SRF ubiquitination, while inhibiting SRF nuclear export (Supplementary Fig. 13d–f). These results indicate that SIRT7 activation by NMN supplementation protects against aneurysm formation.

**Fig. 8:**
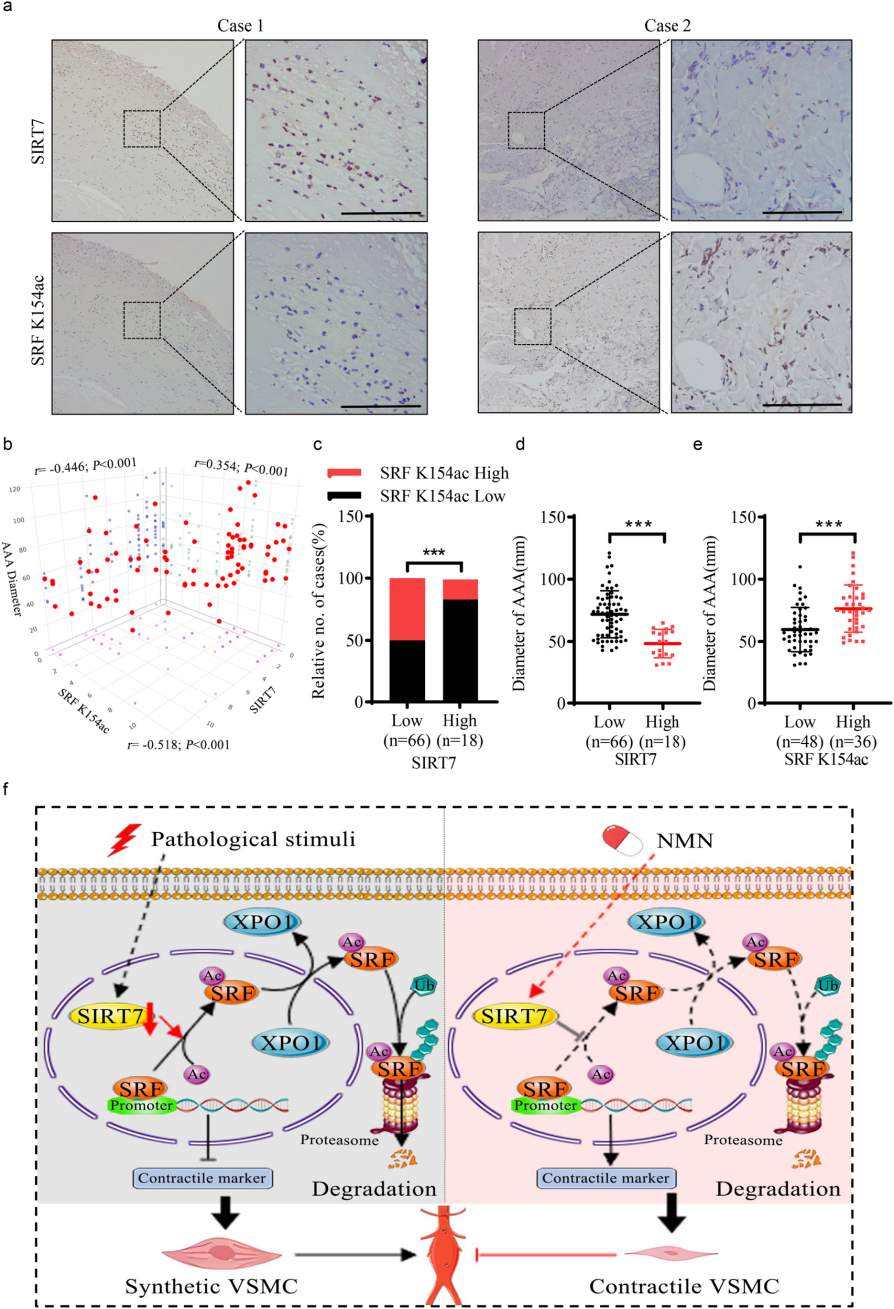
SRF-K154 acetylation is upregulated in patients with AAA and negatively correlated with AAA diameter. **a** Representative image of immunohistochemical (IHC) staining for sirtuin 7 (SIRT7) and serum response factor (SRF)-K154 acetylation in patients with abdominal aortic aneurysm (AAA). Scale bar, 100 μm. **b** Scatter plot of the IHC staining scores for SIRT7, SRF-k154ac, and diameter of AAA in patients (*n* = 84). **c** Quantitative IHC staining scores showing the correlation between SIRT7 and SRF-K154 acetylation. **d-e** The diameter of AAA in indicated groups according to IHC staining scores. **f** Schematic representation of the molecular mechanisms. Under pathological conditions of AAA formation, downregulation of SIRT7 increases SRF acetylation, suppresses SRF transcriptional activity, and promotes XPO-1-mediated SRF nuclear–cytoplasmic translocation, ultimately accelerating SRF ubiquitination and degradation. These events trigger vascular smooth muscle cell (VSMC) phenotypic switching, leading to AAA progression. Supplementation with nicotinamide mononucleotide (NMN), a precursor of NAD^+^ biosynthesis, activates SIRT7, inhibits VSMC phenotypic switching, and attenuates AAA formation. Data are expressed as mean ± SD. All *P*-values and *r*-values were calculated with Spearman’s *r* test. Fisher’s exact test (part **c**). Two-tailed paired Student’s *t* test (parts **d** and **e**).

To investigate the clinical significance of SIRT7-SRF deacetylation during AAA formation, immunohistochemistry staining of SIRT7 and SRF-K154 acetylation was performed and correlated with AAA diameter. Data suggested that SIRT7 expression negatively correlated with SRF acetylation and aortic diameter, whereas SRF acetylation positively correlated with aneurysm size (Fig. 8a–e and Supplementary Table 3). These findings were echoed by the murine AAA model, showing that SIRT7 deletion increased SRF-K154 acetylation and decreased SRF protein abundance, whereas SIRT7 overexpression restored both (Supplementary Fig. 15a–c). Collectively, our data underscore that SIRT7-mediated SRF-K154 deacetylation inhibits SRF nuclear export and ubiquitination-mediated degradation, preserves VSMC contractility, and prevents AAA formation. Pharmacological activation of SIRT7 by NMN may thus represent a promising therapeutic approach for AAA (Fig. 8f).

## Discussion

AAA is a fatal vascular disease characterized by a high risk of rupture; however, no effective pharmacological treatments have been approved to prevent progression^39^. In this study, we identify SIRT7 as a previously unrecognized endogenous protective factor and demonstrate that SIRT7 suppresses aneurysm formation by maintaining the VSMC contractile phenotype through site-specific deacetylation of SRF and that activation of SIRT7 using the NAD⁺ precursor NMN significantly attenuates aortic dilation, establishing a mechanistically grounded and potentially translatable therapeutic strategy for AAA.

The HDAC family has a regulatory role in epigenetic and non-epigenetic signaling via deacetylation of histone and non-histone substrates. HDACs have been developed or tested for tumors, cardiovascular diseases, and metabolic disorders^40^. In recent years, several HDAC family members have been reported to exhibit altered expression in AAA. Specifically, HDAC2, HDAC4, HDAC5, and HDAC7 are upregulated in AAA, whereas SIRT1 and SIRT6 are downregulated^9,41^. However, conflicting findings exist. For example, Xia et al.^10^ reported HDAC1 upregulation in regulatory T cells, whereas Jiang et al.^42^ observed HDAC1 downregulation in T cells in human AAA. Pharmacological studies have shown that pan-HDAC inhibitors can alleviate AngII-induced AAA^12^, whereas Zhang et al.^14^ found that panobinostat, a pan-HDAC inhibitor, exacerbated β-aminopropionitrile-induced thoracic aortic aneurysm. These discrepancies highlight isoform-dependent complexity of HDACs in AAA pathogenesis, underscoring the need for systematic, isoform-specific investigation. In this study, our expression profiling of 18 HDACs in human AAA tissues confirmed the marked upregulation of HDAC2, consistent with previous reports^9^, whereas SIRT7 showed a significant but more modest reduction. As HDACs have been relatively well characterized in AAA and SIRT7 represents an unexplored regulator, and our preliminary studies suggested a VSMC phenotypic switching upon SIRT7 knockdown, we focussed on protective effect of SIRT7 on AAA. By systematically profiling all HDAC family members, we demonstrate that SIRT7 deficiency directly promotes aneurysm development, and that RNA sequencing revealed destruction of VSMC contractile gene programs, identifying SIRT7 as a key regulator of phenotypic stability. These results provide compelling evidence for the previously unrecognized role of VSMC-derived SIRT7 as a positive regulator in protecting against aneurysm formation.

SIRT7 has been demonstrated to regulate inflammation, cell survival, and smooth muscle function in the pathophysiology of various cardiovascular diseases^21,43,44^. Although global SIRT7 deficiency is known to cause systemic phenotypes such as cardiac hypertrophy and metabolic alterations, a VSMC-specific knockout model circumvented these systemic confounders, revealing a direct protective role of SIRT7 in vascular remodeling. In this study, through transcriptomics analysis and VSMC-specific knockout models, we identify VSMC phenotypic control as the primary mechanism underlying SIRT7-mediated aneurysm protection. Given the established deacetylase activity of SIRT7, we used catalytic mutants to investigate its function. Our results demonstrate that the protective effects of SIRT7 strictly depend on its deacetylase activity. Subsequently, MS analysis identified SRF, the master transcription factor for the VSMC contractile phenotype, as a direct downstream interacting protein of SIRT7. Direct deacetylation at SRF-K154 by SIRT7 facilitated contractile gene expression and maintenance of the VSMC contractile phenotype. Interestingly, Zhong et al.^45^ reported that HDAC3 deacetylates SRF, reducing its transcriptional activity and inhibiting carotid artery neointima formation. However, we observed no difference in HDAC3 expression in AAA, suggesting that distinct HDAC–SRF regulatory axes operate in different vascular pathologies. Moreover, we demonstrate that SRF acetylation at K154 drives XPO1-dependent nuclear export and ubiquitin-mediated degradation, identifying a previously unrecognized post-translational mechanism governing SRF stability. These data provide new evidence supporting the role of SIRT7-mediated SRF-K154 deacetylation in maintaining the VSMC contractile phenotype and inhibiting AAA formation.

NMN, a precursor of NAD^+^, serves as an essential co-substrate for NAD^+^-dependent enzymes. As a clinically relevant metabolic intermediate, NMN has been evaluated in clinical trials for its therapeutic potential in treating age-related diseases^46,47^. Prior studies demonstrated that NMN activates SIRT7, offering protective effects in multiple cardiovascular diseases, such as pulmonary hypertension, hypertrophic cardiomyopathy, and vascular aging^48–50^. In this study, exogenous NMN supplementation significantly ameliorated VSMC phenotypic switching and reversed aortic dilation in both AngII-induced and CaCl_2_-induced AAA models. Our data provide valuable insights into the therapeutic potential of NMN in AAA prevention and treatment. We acknowledge that NMN is not a SIRT7-specific activator. At the dose used in this study, NMN may exert nonspecific effects by elevating NAD^+^ levels and consequently activating multiple NAD^+^-dependent enzymes, including other sirtuin family numbers. Nevertheless, our genetic loss-of-function and gain-of-function experiments consistently support SIRT7 as a key mediator of protective effects of NMN in VSMC phenotype maintenance and AAA pathogenesis, although there may be other parallel pathological mechanisms independently contributing to AAA. Regarding safety, according to the FDA guidelines^51^ and widely accepted formulas for animal-to-human dose translation^52^, the 500 mg/kg dose used in mice translates to a human equivalent dose of ~2,430 mg/day, which is below the threshold associated with gastrointestinal and hepatic toxicity and is supported by favorable safety profiles in preclinical and clinical studies^53^.

Despite these compelling and promising findings, several limitations should be acknowledged. First, although multiple mouse models were used, murine AAA does not fully recapitulate the complex genetic, environmental, and hemodynamic factors present in human disease. This may lead to an overestimation of the magnitude of SIRT7’s protective effects when extrapolated to patients. Second, the sample size of human AAA tissues was relatively modest, which may introduce sampling bias and limit the generalizability of the observed associations among SIRT7 expression, SRF acetylation, and aortic diameter; this bias could shift the observed correlations in either direction depending on patient selection. Third, although our data indicate that NMN exerts protective effects partly via SIRT7 activation, NMN is not SIRT7-specific and may influence other sirtuins or NAD⁺-dependent pathways; such off-target effects could inflate the perceived therapeutic benefit attributable solely to SIRT7. Fourth, although we have demonstrated a causal link between SIRT7-mediated SRF deacetylation and VSMC phenotypic maintenance in experimental systems, other indirect pathways and systemic effects cannot be fully excluded; these unmeasured confounders may result in either overestimation or underestimation of the true effect size. Fifth, only male mice were included, which preclude assessment of sex-specific differences in SIRT7 function in AAA. Sixth, the control human tissues used in this study were derived from adjacent relatively normal aortas of patients with AAA; incorporating aortas from non-AAA organ donors will be necessary to further validate our findings. Finally, in our in vivo models, NMN was administered before AAA induction, which primarily highlights its preventive rather than curative potential, exploring that post-disease establishment models are needed to assess the efficacy of NMN in reversing advanced AAAs. Future work should address these limitations by validating our findings in larger, sex-balanced human cohorts, employing more physiologically relevant models, and developing SIRT7-specific pharmacological activators to minimize off-target effects. Thus, more comprehensive and in-depth studies are needed to elucidate the molecular functions of SIRT7, which will facilitate the development of more specific activators for AAA treatment.

In conclusion, we identify SIRT7 as a potential central regulator of AAA pathogenesis. Our study established SRF as a substrate and demonstrated that SIRT7-mediated deacetylation of SRF-K154 is essential for SRF transcriptional activity and protein stability. These findings uncover a conserved signaling axis governing aortic integrity. Furthermore, NAD⁺ augmentation emerges as a rational non-surgical therapeutic approach. These findings advance understanding of AAA biology and highlight the SIRT7–SRF axis as a promising therapeutic target. Future work should focus on developing selective SIRT7 activators and testing their safety and efficacy in preclinical and clinical settings.

## Supplementary information

Supplementary information

Supplementary Data 1

Supplementary Data 2

## Data Availability

All data are available in the main text or in the supplementary materials. All sequencing data generated in this study have been deposited in the NCBI’s Gene Expression Omnibus (GEO) repository (GSE 301109). Unprocessed images are deposited at Mendeley and can be accessed at 10.17632/ztb4vd256g.1. All deposited data will be publicly available upon publication. Any additional information required to reanalyze the data reported in this paper will be shared on reasonable request to the corresponding author.
