# Supplementary material for: Deacetylation of SRF catalyzed by SIRT7 protects against abdominal aortic aneurysm formation
Source: Exp Mol Med. 2026 Jul 8;58(7):2308–24. doi: 10.1038/s12276-026-01787-z (PMC13434582; doi:10.1038/s12276-026-01787-z)

SUPPLEMENTAL MATERIAL

Deacetylation of SRF catalyzed by SIRT7 protects against abdominal aortic aneurysm formation

Short Title: *Xiong et al.; SIRT7 deacetylated SRF inhibiting AAA*

Yacheng Xiong[1]; Jing Chen[2]; Weiting Lu[1]; Yu Liu[1]; Chang sheng[1]; Shuai Liu[1]; Baohui Xu,[4] Yongqing Li,[5] Baihong Pan[1 *]; Wei Wang[1,2,3 *].

1 Department of General &Vascular Surgery, Xiangya Hospital, Central South University, Changsha 410008, China

2 National Clinical Research Center for Geriatric Disorders, Xiangya Hospital, Central South University, Changsha 410008, China

3 Clinical Research Center for Vascular Intervention in Hunan Province, Xiangya Hospital, Central South University, Changsha 410008, China

4 Department of Surgery, Stanford University School of Medicine, Stanford, Calif

5 Department of Surgery, University of Michigan Medical School, Ann Arbor, MI, USA

***Correspondence to**:

Baihong Pan: pbh1990s@csu.edu.cn; Wei Wang: weiwangcsu@csu.edu.cn

Address correspondence to: General &Vascular Surgery, Xiangya Hospital, Central South University, 87#, Xiangya Road, Changsha 410008, Hunan Province, China

The PDF file includes:

Expanded Methods

Supplementary Fig. 1-15

Supplementary Table 1-4

Other Supplementary Material for this manuscript includes the following:

Supplementary Data 1 and 2

Expanded Methods

Weight, Blood pressure and Lipid measurements

Body weights were recorded using a high-precision electronic scale (Model XYZ, Company) at baseline (28 days pre-AngII) and 28 days post-AngII perfusion. Systolic blood pressure was measured by non-invasive tail-cuff plethysmography (BP-2010A, Softron Biotechnology) before and after AngII perfusion. At 28 days post-perfusion, tail vein blood samples were analyzed for triglycerides and total cholesterol using a commercial lipid analyzer (PFS-30, Youlite Medical Technology Co., Ltd, Guilin, China).

Aortic ultrasound imaging

Abdominal aortic ultrasound imaging was performed on isoflurane-anesthetized mice using a Mindray Resona 7 system equipped with ZS3 Exp Domain technology. Cross-sectional views were analyzed for end-diastolic maximal lumen diameters, while longitudinal views assessed morphological characteristics. All measurements were conducted independently by two blinded operators.

Nuclear and Cytoplasmic Extraction

The cytoplasmic and nuclear fractions were isolated using Nuclear and Cytoplasmic Protein Extraction Kit (P0027 Beyotime) in accordance with the manufacturer's instruction. Briefly, cell samples were collected and washed once with ice-cold PBS. Cell pellet was resuspended with reagent A containing protease inhibitor. After 15-min ice incubation, Reagent B was added to lyse plasma membranes under hypotonic conditions while maintaining nuclear integrity.

Then cytoplasmic fraction (supernatant) from the nuclei (pellet) was separated at 4℃, 2000 rpm for 5 mins. Nuclear proteins were extracted by resuspending pellets in lysis buffer. All extracts were stored at -80℃. Fractionation efficiency was verified by GAPDH (cytoplasmic marker) and histone H3 (nuclear marker).

Hematoxylin and eosin (HE) staining

The arterial tissue was embedded in paraffin after fixed with 4% paraformaldehyde (PFA). The arterial sections (5μm) were deparaffinized, rehydrated, and stained with hematoxylin (nuclear staining). After bluing with ammonia water, counterstaining was performed with eosin (cytoplasmic staining). Histological morphology was evaluated using a Leica DM6 M microscope (Leica Microsystems).

Verhoeff Van Gieson (EVG) staining

The aorta tissues were harvested and fixed with 4% PFA before paraffin embedding. Tissue sections (5μm) were stained for elastic fibers using a Verhoeff-Van Gieson (VVG) Elastic Stain Kit (Leagene, DC0059) according to the manufacturer's protocol. Images were captured with a Leica DM6 M microscope. The severity of elastin fragmentation was evaluated and graded as follows: Grade 1 (mild, <25% loss), Grade 2 (moderate, 25–50%), Grade 3 (severe, 50–75%), and Grade 4 (extensive, >75%). Two blinded pathologists independently evaluated all sections, with discordant cases resolved through re-examination.

Immunohistochemistry (IHC) Staining

The clinical aorta tissues were fixed in 4% paraformaldehyde, embedded in paraffin and sectioned (5 μm). Antigen retrieval was performed with Sodium citrate-EDTA buffer by autoclaving (6 min), followed by blocking nonspecific binding with 3% BSA containing 0.3% Triton X-100 for 1 hour. Tissue sections were incubated with primary antibodies overnight at 4°C, followed by HRP-conjugated secondary antibodies and DAB development (ZSGB-BIO, ZLI-9018). Hematoxylin counterstaining was applied. Images were obtained by a Leica DM6 M microscope. Two experienced pathologists independently received and scored immunostaining results. The immune response score was used to quantify IHC results. The percentage scores for positive stained areas were as follows: 1 (<10%), 2 (10%-50%), 3 (50-75%), and 4 (> 75%). The staining intensity score was as follows: 0 = no staining; 1 = mild staining; 2 = moderate staining; 3 = strong staining. The final IHC score (range: 0–12) was calculated by multiplying both scores, with ≥9 defined as high expression. The antibodies used can be obtained in Supplementary Table 1.

Immunofluorescence staining

For immunofluorescence, tissue sections were deparaffinized, rehydrated, and subjected to antigen retrieval. Cells were fixed with 4% PFA (15 min), permeabilized with 0.3% Triton X-100 (10 min), and blocked with 3% BSA (1 h, RT). Samples were incubated with primary antibodies (4°C, overnight) followed by fluorophore-conjugated secondary antibodies (1 h, RT). Nuclei were stained with DAPI (SouthernBiotech, 0100-20, 5 min). The antibodies used are listed in supplemental Supplementary Table 1. Tissue images were obtained by a Leica DMi8

M (Leica Microsystems, Germany), while cell images were acquired on a LSM 880 confocal microscope (Carl Zeiss AG, Germany).

Coomassie blue staining

Protein gel was fixed in a fixation solution to precipitate proteins and remove SDS after electrophoresis. After staining with Coomassie Brilliant Blue Fast staining solution (P0017, Beyotime) for 30 min, excess dye was removed by destaining in deionized water. Gel images were captured using a ChemiDoc MP system (Bio-Rad). Gel strips can be used for mass spectrometric analysis.

Western Blot

Samples were harvested and lysed with NP-40 or RIPA containing protease and phosphatase Inhibitor cocktails. Cell lysates were centrifuged at 4℃ 12000 rpm for 15 min and protein concentration in the supernatant was determined with the bicinchoninic acid protein assay (BCA, P0011, Beyotime). Protein samples were diluted with 5x SDS loading buffer and boiled at 95℃ for 10 min. Then proteins were separated by electrophoresis and transferred onto a PVDF membrane. The membranes were incubated with primary antibodies overnight at 4℃ after blocking with 5% skim milk, followed by incubation with HRP-conjugated secondary antibodies for 1h at room temperature. Then, Signals were detected with ECL reagent (Millipore, Billerica, MA, USA). Immunoblots were analyzed with Image J software. Antibodies utilized in this study are listed in Supplementary Table 1.

PCR

Total RNA of tissue or cell sample was extracted by TRIzol reagent method. Total RNA was used for first-strand cDNA synthesis with Hifair® AdvanceFast One-step RT-gDNA Digestion SuperMix (11151ES60, Yeasen). qRT-PCR was performed utilizing 2X Universal SYBR Green Fast qPCR Mix (RK21203, ABclonal) on the Applied Biosystems ViiA 7 Real-Time PCR System (ThermoFisher Scientific). PCR conditions included initial denaturation at 95°C for 5 min, annealing at 95°C for 5s and extension at 60°C for 31s for 40 cycles. Gene expression levels were normalized to GAPDH. Relative gene expression was determined by the $2^{-\Delta\Delta Ct}$ method. The primers of genes are listed in Supplementary Table 4.

Supplementary Fig. 1. SIRT7 knockout aggravated AngII-induced AAA formation in vivo.

(**a**) Schematic diagram of construction strategies of Sirt7 knockout (*Sirt7*$^{-/-}$) mice.

(**b**) Genotyping was detected by tail preparation of *ApoE*$^{-/-}$*Sirt7*$^{-/-}$ breeding mice.

(**c**) Measurement of SIRT7 protein in arterial tissue was reflected the knockout effect in *ApoE*$^{-/-}$*Sirt7*$^{-/-}$ mice. n=3.

(**d**) Representative ultrasonic images and quantitative comparison of maximal lumen diameter in *ApoE*$^{-/-}$*Sirt7*$^{+/+}$ and *ApoE*$^{-/-}$*Sirt7*$^{-/-}$ mice after 28 days of AngII infusion. n=10 for saline and n=20 for AngII. Scale bar: 1 mm

(**e**) Representative macroscopic images and quantitative comparison of maximal aortic diameter in *ApoE*$^{-/-}$*Sirt7*$^{+/+}$ and *ApoE*$^{-/-}$*Sirt7*$^{-/-}$ mice after 28 days of AngII infusion. n=10 for saline and n=20 for AngII.

(**f**) Survival curves of AngII-induced AAA in indicated groups. n=20 per group.

(**g-h**) The incidence and rupture rate of AngII-induced AAA in indicated groups. n=20 per group.

(**i**) Representative HE and VVG staining images and quantitative comparison of VVG staining score in *ApoE*$^{-/-}$*Sirt7*$^{+/+}$ and *ApoE*$^{-/-}$*Sirt7*$^{-/-}$ mice after AngII infusion. n = 8. Scale bars, 100 μm.

(**j-k**) Relative mRNA and protein expression of SMC contractile marker (*Myh11*, *Acta2*, *Cnn1* and *Tagln*) in *ApoE*$^{-/-}$*Sirt7*$^{+/+}$ and *ApoE*$^{-/-}$*Sirt7*$^{-/-}$ mice after 28 days of AngII infusion. n=6 for mRNA, n=3 for protein.

(**l-m**) Systolic blood pressure and Body weight before and 28 days after AngII infusion in *ApoE*$^{-/-}$*Sirt7*$^{+/+}$ and *ApoE*$^{-/-}$*Sirt7*$^{-/-}$ mice. n=20 per group.

(**n**) Total cholesterol (TC) and triglycerides (TG) in AngII-induced *ApoE*$^{-/-}$*Sirt7*$^{+/+}$ and *ApoE*$^{-/-}$

Sirt7$^{-/-}$ mice. n=15 for *ApoE*$^{-/-}$*Sirt7*$^{+/+}$ and n=10 for *ApoE*$^{-/-}$*Sirt7*$^{-/-}$.

Data are expressed as mean ± SD. (**d, e, k, l**, **m, n**) Two-way ANOVA followed by Bonferroni multiple comparisons. (**f**) Kaplan-Meier method followed by log-rank tests. (**g, h**) Fisher exact test. (**i**) Two-tailed unpaired student's t test.

Supplementary Fig. 2. SIRT7 knockout aggravated $CaCl_2$-induced AAA formation in vivo

(**a**) Schematic diagram of the $CaCl_2$-induced AAA model in *Sirt7*$^{+/+}$ and *Sirt7*$^{-/-}$ mice.

(**b**) Representative ultrasonic images and quantitative comparison of maximal lumen diameter in *Sirt7*$^{+/+}$ and *Sirt7*$^{-/-}$ mice in $CaCl_2$-induced AAA. n=8 for saline and n=12 for $CaCl_2$. Scale bar: 1 mm.

(**c**) Representative HE and VVG staining images and quantitative comparison of VVG staining score in $CaCl_2$-induced *Sirt7*$^{+/+}$ and *Sirt7*$^{-/-}$ mice. n = 8. Scale bars, 50 μm.

(**d**) Representative macroscopic images and quantitative comparison of maximal aortic diameter in $CaCl_2$-induced *Sirt7*$^{+/+}$ and *Sirt7*$^{-/-}$ mice. n=8 for saline and n=12 for $CaCl_2$.

Data are expressed as mean ± SD. (**b** and **d**) Two-way ANOVA followed by Bonferroni multiple comparisons. (**c**) Two-tailed unpaired student's t test.

Supplementary Fig. 3. Parameters of the AngII-induced AAA model in VSMC-specific SIRT7 knockout mice

(**a**) Schematic diagram of construction strategies of VSMC-specific Sirt7 knockout (*Sirt7*$^{\Delta SMC}$) mice.

(**b**) Genotyping was detected by tail preparation of *ApoE*$^{-/-}$*Sirt7*$^{\Delta SMC}$ breeding mice.

(**c**) Measurement of SIRT7 protein in arterial smooth muscle layer was reflected the knockout effect in *ApoE*$^{-/-}$*Sirt7*$^{\Delta SMC}$ mice. n=3.

(**d**) Representative immunofluorescence staining images of Sirt7 in *ApoE*$^{-/-}$*Sirt7*$^{\Delta SMC}$ mice abdominal aorta. Scale bar: 100μm.

(**e-f**) Systolic blood pressure and Body weight before and 28 days after AngII infusion in *ApoE*$^{-/-}$*Sirt7*$^{flox/flox}$ and *ApoE*$^{-/-}$*Sirt7*$^{\Delta SMC}$ mice. n=20 per group.

(**g**) Total cholesterol (TC) and triglycerides (TG) in AngII-induced *ApoE*$^{-/-}$*Sirt7*$^{flox/flox}$ and *ApoE*$^{-/-}$*Sirt7*$^{\Delta SMC}$ mice. n=16 for *ApoE*$^{-/-}$*Sirt7*$^{flox/flox}$ and n=11 for *ApoE*$^{-/-}$*Sirt7*$^{\Delta SMC}$.

(h) Representative HE and VVG staining images and quantitative comparison of VVG staining score in ApoE-/-Sirt7flox/flox and ApoE-/-Sirt7ΔSMC mice after AngII infusion. n = 8. Scale bars, 100 μm.

Data are expressed as mean ± SD. (**e**, **f, g**) Two-way ANOVA followed by Bonferroni multiple comparisons. (**h**) Two-tailed unpaired student's t test.

Supplementary Fig. 4. VSMC-specific SIRT7 deficiency aggravated $CaCl_2$-induced AAA formation in vivo

(**a**) Schematic diagram of the $CaCl_2$-induced AAA model in *Sirt7*$^{flox/flox}$ and *Sirt7*$^{\Delta SMC}$ mice.

(**b**) Representative ultrasonic images and quantitative comparison of maximal lumen diameter in *Sirt7*$^{flox/flox}$ and *Sirt7*$^{\Delta SMC}$ mice in $CaCl_2$-induced AAA. n=8 for saline and n=12 for $CaCl_2$. Scale bar: 1 mm.

(**c**) Representative HE and VVG staining images and quantitative comparison of VVG staining

score in $CaCl_2$-induced *Sirt7*$^{+/+}$ and *Sirt7*$^{-/-}$ mice. n = 8. Scale bars, 100 μm.

(**d**) Representative macroscopic images and quantitative comparison of maximal aortic diameter in $CaCl_2$-induced *Sirt7*$^{flox/flox}$ and *Sirt7*$^{\Delta SMC}$ mice. n=8 for saline and n=12 for $CaCl_2$.

Data are expressed as mean ± SD. (**b** and **d**) Two-way ANOVA followed by Bonferroni multiple comparisons. (**c**) Two-tailed unpaired student's t test.

Supplementary Fig. 5. SIRT7 overexpression repress AngII-induced AAA formation in vivo

(**a**) Relative SIRT7 mRNA and protein expression were determined in *ApoE*$^{-/-}$ mice treated with AAV-EV or AAV-*Sirt7*. n=6 for mRNA, n=3 for protein.

(**b**) Representative HE and VVG staining images and quantitative comparison of VVG staining score in indicated groups. n = 8. Scale bars, 100 μm.

(**c-d**) Systolic blood pressure and Body weight before and 28 days after AngII infusion in AAV-EV and AAV-*Sirt7* mice. n=20 per group.

(**e**) Total cholesterol (TC) and triglycerides (TG) in AngII-induced *ApoE*$^{-/-}$ mice treated with AAV-EV or AAV-*Sirt7*. n=14 for AAV-EV and n=18 for AAV-*Sirt7*.

Data are expressed as mean ± SD. (**a, b**) Two-tailed unpaired student's t test. (**c, d, e**) Two-way ANOVA followed by Bonferroni multiple comparisons.

Supplementary Fig. 6. SIRT7 regulates VSMC phenotypic switching in vitro

(**a**) HASMC was serum-free cultured for 24h, and then treated with PDGF-BB (20 ng/mL) for 24h. Relative mRNA of *SIRT7*, *MYH11*, *ACTA2*, *CNN1* and *TAGLN* was determined by qPCR.

n=3.

(**b**) HASMC was cultured in complete medium for 24h, and then treated with serum-starved for 24h. Relative mRNA of *SIRT7*, *MYH11*, *ACTA2*, *CNN1* and *TAGLN* was determined by qPCR. n=3.

(**c**) HASMC was infected with lentivirus containing shNC or shSIRT7. After 48h, the cells were serum-free cultured for 24h. The protein expression of SIRT7 was determined by WB. n=3.

(**d**) HASMC was infected with lentivirus containing empty vector (EV) or Flag-SIRT7. After 48h, the cells were treated with PDGF-BB (20 ng/mL) for 24h. The protein expression of SIRT7 was determined by WB. n=3.

(**e**) Protein quantification of SIRT7, MYH11, α-actin, Calponin1 and SM22α was detected in HASMC with different treatments as described in (C) and (D). n=3.

(**f**) mRNA expression of SIRT7, MYH11, ACTA2, CNN1 and TAGLN was detected in HASMC with different treatments as described in (C) and (D). n=3.

(**g** and **h**) mRNA and protein expression of Sirt7, Myh11, Acta2, Cnn1 and Tagln was detected in MASMC with different treatments as described in (C) and (D). n=3.

Data are expressed as mean ± SD. (**a, b, e, f, g, h**) Two-way ANOVA followed by Bonferroni post hoc test.

Supplementary Fig. 7. SIRT7 regulate VSMC phenotypic switching depends on its deacetylation

(**a** and **b**) HASMC was infected with lentivirus containing EV, SIRT7-WT or SIRT7-H187Y.

After 48h, the cells were treated with PDGF-BB (20 ng/mL) for 24h. The mRNA and protein expression of SIRT7, MYH11, ACTA2, CNN1 and TAGLN was determined by qPCR and WB. n=4.

(**c** and **d**) mRNA and protein expression of SIRT7, MYH11, ACTA2, CNN1 and TAGLN was detected in MASMC with different treatments as described in (A) and (B). n=4.

Data are expressed as mean ± SD. (**b** and **d**) One-way ANOVA followed by Bonferroni post hoc test.

Supplementary Fig. 8. SIRT7 directly interacts with SRF and regulates the deacetylation of SRF

(**a**) LC-MS/MS spectrum showing the SRF peptides pulled down by SIRT7.

(**b**) Co-IP of exogenous Flag-SIRT7 and HA-SRF in HEK293T.

(**c**) Co-IP of endogenous SIRT7 and SRF in MASMC.

(**d**) Protein quantification of SIRT7, SRF, MYH11, α-actin, Calponin1 and SM22α was detected in HASMC with different treatments as described in Fig. 3G. n=3.

(**e**) HASMC was infected with lentivirus containing EV or HA-SRF. After 48h, the cells were treated with PDGF-BB (20 ng/mL) for 24h. Then WCE was collected for IP with anti-HA antibody magnetic beads, followed by WB.

(**f**) HASMC was infected with lentivirus containing shNC, shSIRT1, shSIRT6 and shSIRT7 for 48h, followed by serum starvation for 24h. WCE was collected for IP with anti-SRF antibody, followed by WB.

(**g**) Protein quantification of Pan-Kac, HA-SRF was detected in HASMC with different

treatments as described in Fig. 3K. n=3.

(**h**) SRF K154ac site in different species.

(**i**) Different peptides of SRF K154 (0.1 – 1 μg) was added onto the PVDF membranes, followed by WB analysis using a K154-specific acetylation antibody (anti-SRF K154ac).

(**j**) WB analysis of SRF expression level in SRF-WT and SRF-KO A7r5.

(**k**) WB analysis of SRF expression level in A7r5$^{SRF-/-}$ expressing ectopic SRF-WT, SRF K150R or SRF K150Q.

(**l-n**) Protein quantification of SIRT7, SRF, MYH11, α-actin, Calponin1 and SM22α was detected in A7r5$^{SRF-/-}$ with different treatments as described in Fig. 3P-R. n=3.

All immunoblotting was independently performed 3 times. Data are expressed as mean ± SD. (**d, l, m, n**) Two-way ANOVA followed by Bonferroni post hoc test. (**g**) One-way ANOVA followed by Bonferroni post hoc test.

Supplementary Fig. 9. SIRT7 modulates nucleoplasmic translocation and ubiquitination of SRF

(**a** and **b**) MASMC was infected with lentivirus containing shNC, shSIRT7 or EV, SIRT7-WT, SIRT7-H188Y. After 48h, the cells were serum-starved or treated with PDGF-BB (20 ng/mL) for 24h and then treated with CHX (10 μg/mL) for 0, 3, 6, and 12h. WCE was collected for WB and remaining of SRF expression was calculated. n=3.

(**c**) Protein quantification of SIRT7, SRF was detected in HASMC with different treatments as described in Fig. 4D. n=3.

(**d-e**) Protein quantification of α-ub, SRF-K154ac was detected in A7r5$^{SRF-/-}$ with different

treatments as described in Fig. 4E-4F. n=3.

(**f** and **g**) MASMC was infected with lentivirus containing shNC, shSIRT7 or EV, SIRT7-WT, SIRT7-H188Y. After 48h, the cells were treated with PDGF-BB (20 ng/mL) for 24h. WCE was collected for IP with SRF antibody followed by WB analysis. n=3.

Data are expressed as mean ± SD. (**a-e**) Two-way ANOVA followed by Bonferroni post hoc test.

Supplementary Fig. 10. The nuclear translocation of SRF is dependent on XPO1

(**a**) Protein quantification of α-ub, HA-SRF was detected in HASMC with different treatments as described in Fig. 5A. n=3.

(**b-c**) Protein quantification of HA-SRF was detected in HASMC with different treatments as described in Fig. 5B-5C. n=3.

(**d**) Cytosolic and nuclear fractions from A7r5$^{SRF-/-}$ overexpressed HA-SRF or mutant were collected and subjected to WB analysis.

(**e**) Nuclear fractions from A7r5$^{SRF-/-}$ infected with HA-SRF or HA-SRF-K150R/Q mutants and treated with PDGF-BB (20 ng/mL) were collected and subjected to WB analysis.

(**f** and **g**) Cytosolic and nuclear fractions from MASMC silenced with shNC, shSIRT7 or overexpressed EV, SIRT7 were collected and subjected to WB analysis.

(**h-i**) Protein quantification of SRF was detected in HASMC with different treatments as described in Fig. 5E-5F. n=3.

(**j**) Nuclear fractions from MASMC silenced with shXPO1, shXPO4, shXPO5 or shXPO7 and treated with PDGF-BB (20 ng/mL) were collected and subjected to WB analysis.

(**k**) Cytosolic and nuclear fractions from MASMC silenced with shXPO1 were collected and subjected to WB analysis.

(**l**) Protein quantification of SRF was detected in HASMC with different treatments as described in Fig. 5H. n=3.

(**m**) Protein quantification of XPO1, SRF was detected in HASMC with different treatments as described in Fig. 5I. n=3.

(**n**) WCE from MASMC overexpressed EV, SIRT7 was collected for IP with SRF antibody, followed by WB analysis.

All immunoblotting was independently performed 3 times. Data are expressed as mean ± SD. (**a-c, h-i, m**) Two-way ANOVA followed by Bonferroni post hoc test. (**l**) One-way ANOVA followed by Bonferroni post hoc test.

Supplementary Fig. 11. SIRT7 stabilizes the transcriptional activity of SRF

(**a** and **b**) MASMC was infected with lentivirus containing shNC, shSIRT7 or EV, SIRT7-WT, SIRT7-H188Y. After 48h, the cells were serum-starved or treated with PDGF-BB (20 ng/mL) for 24h. The cell extracts were collected for CHIP with anti-SRF antibody, followed by qPCR of *Myh11*, *Acta2*, *Cnn1* and *Tagln* gene promoters. n=3.

Data are expressed as mean ± SD. (**a, b**) Two-way ANOVA followed by Bonferroni post hoc test.

Supplementary Fig. 12. Parameters of AAV-*Srf* administration in AngII-infused *ApoE$^{-/-}$ Sirt7$^{\Delta SMC}$* mice

(**a** and **b**) Systolic blood pressure and Body weight before and 28 days after AngII infusion in indicated mice. n=20 per group.

(**c**) Total cholesterol (TC) and triglycerides (TG) in AngII-induced *ApoE*$^{-/-}$*Sirt7*$^{\Delta SMC}$ mice. n=10 for AAV-EV, n=17 for AAV-*Srf-K154R* and n=11 for AAV-*Srf-K154Q*.

Data are expressed as mean ± SD. (**a-c**) Two-way ANOVA followed by Bonferroni post hoc test.

Supplementary Fig. 13. NMN supplementation regulates phenotypic transformation by inhibiting SRF nuclear transfer and ubiquitination

(**a** and **b**) Systolic blood pressure and Body weight before and 28 days after AngII infusion in AngII-induced AAA with saline or NMN administration. n=20 per group.

(**c**) Total cholesterol (TC) and triglycerides (TG) in AngII-induced AAA with saline or NMN administration. n=13 for saline and n=18 for NMN.

(**d**) HASMC was pre-treated with vehicle or NMN (0.5 mM) for 2h, followed by PDGF-BB (20 ng/mL) for 24h. WCE was collected for WB analysis. n=4.

(**e**) WCE from HASMC treated with PDGF-BB (20 ng/mL) after NMN (0.5 mM) administration was collected for IP with SRF antibody, followed by WB analysis. n=3.

(**f**) Cytosolic and nuclear fractions from HASMC treated with PDGF-BB (20 ng/mL) after NMN (0.5 mM) administration were collected and subjected to WB analysis.

Data are expressed as mean ± SD. (**a, b, c**) Two-way ANOVA followed by Bonferroni post hoc test.

Supplementary Fig. 14. Supplementation with Nicotinamide Mononucleotide protects against $CaCl_2$-induced AAA formation

(**a**) Schematic diagram of the $CaCl_2$-induced AAA model in mice administrated with saline or NMN.

(**b**) Representative ultrasonic images and quantitative comparison of maximal lumen diameter in $CaCl_2$-induced mice after NMN administration. n=15 per group. Scale bar: 1 mm

(**c**) Representative macroscopic images and quantitative comparison of maximal aortic diameter in $CaCl_2$-induced mice after NMN administration. n=15 per group.

(**d**) Representative HE and VVG staining images and quantitative comparison of VVG staining score in $CaCl_2$-induced mice after NMN administration. n = 8. Scale bars, 100 μm.

(**e**) Aortic tissues from $CaCl_2$-induced mice after NMN administration were collected for WB analysis. n=3.

Data are expressed as mean ± SD. (**b-d**) Two-tailed paired student's t test.

Supplementary Fig. 15. SIRT7 protects SRF by deacetylating K154 in AngII-induced AAA formation

(**a**) Relative protein expression of SRF K154ac and SRF in *ApoE*$^{-/-}$*Sirt7*$^{+/+}$ and *ApoE*$^{-/-}$*Sirt7*$^{-/-}$ mice after 28 days of AngII infusion. n=3 for protein.

(**b**) Relative protein expression of SRF K154ac and SRF in *ApoE*$^{-/-}$*Sirt7*$^{flox/\mathrm{flox}}$ and *ApoE*$^{-/-}$*Sirt7*$^{\Delta SMC}$ mice after 28 days of AngII infusion. n=3 for protein.

(**c**) Relative protein expression of SMC SRF K154ac and SRF in *ApoE*$^{-/-}$ mice treated with AAV-EV or AAV-*Sirt7* after AngII infusion. n=3 for protein.

Supplementary Table 1: the key reagent and resource used in the present study.

Reagent or Resource	Source	Identifier
**Antibodies**		
Rabbit anti-Sirt7 (H/M/R, WB/IP)	Cell Signaling Technology	Cat# 5360S
Rabbit anti-Sirt7 (H/M, IF/IHC)	Huabio	Cat# HA723075
Mouse anti-SIRT7 (H/M, WB)	Santa Cruz Biotechnology	Cat# sc-365344
Rabbit anti-SRF (H/M/R, WB/IP/CHIP)	Cell Signaling Technology	Cat# 5147S
Rabbit anti-SRF-K154ac (H/M, WB/IHC)	AtaGenix	Cat# 24002720A
Rabbit anti-Exportin1 (XPO1) (H/M, WB/IP)	Cell Signaling Technology	Cat# 46249S
Rabbit anti-Flag (H/M/, WB/IP)	Cell Signaling Technology	Cat# 14793S
Mouse anti- HA (H/M/, WB/IP)	Abclonal	Cat# AE008
Rabbit anti-GST (H, WB)	Abclonal	Cat# AE007
Rabbit anti-Acetylated-Lysine (H/M, WB)	Cell Signaling Technology	Cat# 9441S
Mouse anti-Ubiquitin (H/M, WB)	Cell Signaling Technology	Cat# 3936S
Rabbit anti-Histone H3 (acetyl K9) (H/M, CHIP)	Abcam	Cat# ab32129
Rabbit anti-Histone H3 (acetyl K18) (H/M, CHIP)	Abcam	Cat# ab1191
Rabbit anti-Histone H3 (acetyl K27) (H/M, CHIP)	Abcam	Cat# ab4729
Rabbit anti-smooth muscle Myosin heavy chain 11 (R, WB)	Huabio	Cat# ET1704-61
Rabbit anti-smooth muscle Myosin heavy chain 11 (H/M, WB)	Abcam	Cat# ab124679
Rabbit anti-alpha smooth muscle Actin (H/M/R, WB)	Huabio	Cat# ET1607-53
Rabbit anti-Calponin 1 (H/M/R, WB)	Abcam	Cat# 46794
Rabbit anti-SM22alpha (H/M/R, WB)	Proteintech	Cat# 10493-1-AP
Mouse anti-alpha smooth muscle Actin (H/M, IF)	Abcam	Cat# ab7817
Alexa Fluor 594-conjugated donkey anti-rabbit IgG (H/M, IF)	Invitrogen	Cat# A21207
Alexa Fluor 488-conjugated donkey anti-mouse IgG (H/M, IF)	Invitrogen	Cat# A21202
Rabbit anti-Histone H3 (H/M/R, WB)	Abclonal	Cat# A22348
Rabbit anti-β-Actin (H/M/R, WB)	Abclonal	Cat# AC038
Rabbit anti-GAPDH (H/M/R, WB)	Abcam	Cat# ab181602
**Chemicals**		
Precision Plus Protein Dual Color Standards	Bio-Rad	Cat# 1610374
Prestained Protein Marker VII	Servicebio	Cat# G2087
Protein Marker	Servicebio	Cat# G26619
100 bp DNA Ladder	Beijing Tsingke Biotech Co., Ltd.	Cat# TSJ100-100
Sodium citrate-EDTA antigen repair buffer	Beyotime	Cat# P0086
Mouse/Rabbit Enhanced Polymer test system	ZSGB-BIO	Cat# PV9000
DAB Substrate Kit	ZSGB-BIO	Cat# ZLI-9018

Angiotensin II human	MedChemExpress	Cat# HY-13948
Sterile 0.9% NaCl	Servicebio	Cat# G4702
Opti-MEM	Gibco	Cat# 31985070
β-Nicotinamide mononucleotide	MedChemExpress	Cat# HY-F0004
calcium chloride	Servicebio	Cat# GC102001
Recombinant Human PDGF-BB	Novoprotein	Cat# C199
Recombinant Human TGF-β	Novoprotein	Cat# CA59
Polybrene	Beyotime	Cat# C0351
PEI	MedChemExpress	Cat# HY-K2014
Cycloheximide	MedChemExpress	Cat# HY-12320
MG132	MedChemExpress	Cat# HY-13259
Chloroquine	MedChemExpress	Cat# HY-17589A
DAPI Fluoromount-G	Southernbiotech	Cat# 0100-20
10x Poly-L-lysine Solution	Solarbio	Cat# P2100
RNAiso Plus	Takara	Cat# 9109
RIPA Lysate	Beyotime	Cat# P0013B
NP-40	Beyotime	Cat# P0013F
Protein A/G Magnetic beads	MedChemExpress	Cat# HY-K0202
Anti-Flag Magnetic Beads	MedChemExpress	Cat# HY-K0207
Anti-HA Magnetic Beads	MedChemExpress	Cat# HY-K0201
Anti-GST Magnetic Beads	MedChemExpress	Cat# HY-K0222
Protease and phosphatase inhibitor	Beyotime	Cat# P1045
SDS-PAGE protein loading buffer	Beyotime	Cat# P0015L
Immobilon Western HRP substrate Luminol Reagent	Millipore	Cat# WBKLS0500
Complete Classic Medium With Serum and CultureBoost™	Cell systems	Cat# 4Z0-500
Smooth Muscle Cell Medium	ScienCell	Cat# 1101
Fetal bovine serum	Gibco	Cat# A5670701
penicillin/streptomycin	Gibco	Cat# 15070063
DMEM	Gibco	Cat# C11995500BT
Cell freezing medium	ScienCell	Cat# 0133
**Cultured cell**		
Human aortic smooth muscle cell	Cell systems	Cat# ACBRI 716
Mouse aortic smooth muscle cell	YZFBIO	Cat# YPC-M019
A7r5 cell	YZFBIO	Cat# YCL-0020
HEK293T	ATCC	
**Critical commercial assay**		
TransDirect® Mouse Genotyping Kit	TransGen Biotech Co., Ltd.	Cat# AD501-01
Verheff's Elastic Stain Kit	Leagene	Cat# DC0059
Hifair® AdvanceFast One-step RT-gDNA Digestion SuperMix	Yeasen	Cat# 11151ES60
2X Universal SYBR Green Fast qPCR Mix	Abclonal	Cat# RK21203
Nucleoprotein and cytoplasmic protein	Beyotime	Cat# P0027

extraction kit		
Mut Express II Fast Mutagenesis Kit V2	Vazyme	Cat# C214-01
ChIP Assay Kit	Beyotime	Cat# P2080S
Anti-Flag Affinity Gel	MedChemExpress	Cat# HY-K0217
GST Sepharose 4FF Resin	Sangon Biotech	Cat# C600911
BCA Protein Assay Kit	Beyotime	Cat# P0011
Coomassie Brilliant Blue Fast staining solution	Beyotime	Cat# P0017
2X Universal SYBR Green Fast qPCR Mix	Abclonal	Cat# RK21203
A mini osmotic pump	RWD Life Science Co., Ltd	Cat# 2004W
**Experimental animals**		
C57BL/6J mice	SPF Hunan SJA Laboratory Animal Co., Ltd.	N/A
ApoE$^{-/-}$ mice	SPF Hunan SJA Laboratory Animal Co., Ltd.	N/A
Sirt7$^{-/-}$ mice	Cyagen Biosciences Inc	Cat# S-KO-19934
Sirt7$^{flox/flox}$ mice	Cyagen Biosciences Inc	Cat# S-CKO-17707
Tagln-Cre mice	Cyagen Biosciences Inc	Cat# C001004

Supplementary Table 2: the patient clinical information used in Fig. 1. (n=9)

Patient	Gender	Age	Smoking	Diameter(mm)	HL	HTN	CAD
ID1	Male	69	yes	66	no	yes	no
ID2	Female	63	no	121	no	yes	no
ID3	Male	65	yes	89	yes	no	no
ID4	Male	64	yes	43	yes	no	no
ID5	Male	77	yes	71	yes	yes	no
ID6	Male	58	yes	40	yes	yes	yes
ID7	Male	25	no	68	no	yes	no
ID8	Female	54	no	50	no	no	no
ID9	Female	55	yes	93	no	no	no

HL: Hyperlipidemia; HTN: Hypertension; CAD: Coronary artery disease.

Supplementary Table 3: the patient clinical information used in Fig. 8. (n=84)

	Patients (n=84)
Age (years)	62.85 ± 19.89
Male gender (n, %)	63 (75.0)
Smoking (n, %)	48 (57.1)
Body mass index (kg/m^2)	23.53 ± 2.89
Hyperlipidemia (n, %)	41 (48.8)

Hypertension (n, %)	47 (56.0)
Diabetes Mellitus (n, %)	15 (17.9)
Coronary artery disease (n, %)	19 (22.6)

Data are shown as mean ± standard deviation or frequencies (%).

Supplementary Table 4: The primer sequences used in the present study.

Gene name	Primer Sequence (5'-3')	Application
HDAC1 (H)	Forward: CTACTACGACGGGGATGTTGG	qPCR
	Reverse: GAGTCATGCGGATTCGGTGAG	
HDAC2 (H)	Forward: ATGGCGTACAGTCAAGGAGG	qPCR
	Reverse: TGCGGATTCTATGAGGCTTCA	
HDAC3 (H)	Forward: CCTGGCATTGACCCATAGCC	qPCR
	Reverse: CTCTTGGTGAAGCCTTGCATA	
HDAC4 (H)	Forward: GGCCCACCGGAATCTGAAC	qPCR
	Reverse: GAACTCTGGTCAAGGGAACTG	
HDAC5 (H)	Forward: TCTTGTCGAAGTCAAAGGAGC	qPCR
	Reverse: GAGGGGAACTCTGGTCCAAAG	
HDAC6 (H)	Forward: AAGAAGACCTAATCGTGGGACT	qPCR
	Reverse: GCTGTGAACCAACATCAGCTC	
HDAC7 (H)	Forward: GGCGGCCCTAGAAAGAACAG	qPCR
	Reverse: CTTGGGCTTATAGCGCAGCTT	
HDAC8 (H)	Forward: TCGCTGGTCCCGGTTTATATC	qPCR
	Reverse: TACTGGCCCGTTTGGGGAT	
HDAC9 (H)	Forward: AGTAGAGAGGCATCGCAGAGA	qPCR
	Reverse: GGAGTGTCTTTCGTTGCTGAT	
HDAC10 (H)	Forward: CAGTTCGACGCCATCTACTTC	qPCR
	Reverse: CAAGCCCATTTTGCACAGCTC	
HDAC11 (H)	Forward: ACCCAGACAGGAGGAACCATA	qPCR
	Reverse: TGATGTCCGCATAGGCACAG	
SIRT1 (H)	Forward: TAGCCTTGTCAGATAAGGAAGGA	qPCR
	Reverse: ACAGCTTCACAGTCAACTTTGT	
SIRT2 (H)	Forward: TGCGGAACTTATTCTCCCAGA	qPCR
	Reverse: GAGAGCGAAAGTCGGGGAT	
SIRT3 (H)	Forward: ACCCAGTGGCATTCCAGAC	qPCR
	Reverse: GGCTTGGGGTTGTGAAAGAAG	
SIRT4 (H)	Forward: GCTTTGCGTTGACTTTCAGGT	qPCR
	Reverse: CCAATGGAGGCTTTCGAGCA	

SIRT5 (H)	Forward: GCCATAGCCGAGTGTGAGAC	qPCR
	Reverse: CAACTCCACAAGAGGTACATCG	
SIRT6 (H)	Forward: CCCACGGAGTCTGGACCAT	qPCR
	Reverse: CTCTGCCAGTTTGTCCCTG	
SIRT7 (H)	Forward: GACCTGGTAACGGAGCTGC	qPCR
	Reverse: CGACCAAGTATTTGGCGTTCC	
MYH11 (H)	Forward: CGCCAAGAGACTCGTCTGG	qPCR
	Reverse: TCTTTCCCAACCGTGACCTTC	
ACTA2 (H)	Forward: AAAAGACAGCTACGTGGGTGA	qPCR
	Reverse: GCCATGTTCTATCGGGTACTTC	
CNN1 (H)	Forward: CTGTCAGCCGAGGTTAAGAAC	qPCR
	Reverse: GAGGCCGTCCATGAAGTTGTT	
TAGLN (H)	Forward: AGTGCAGTCCAAAATCGAGAAG	qPCR
	Reverse: CTTGCTCAGAATCACGCCAT	
SRF (H)	Forward: CGAGATGGAGATCGGTATGGT	qPCR
	Reverse: GGGTCTTCTTACCCGGCTTG	
MYOCD (H)	Forward: ACGGATGCTTTTGCCTTTGAA	qPCR
	Reverse: AACCTGTCGAAGGGGTATCTG	
MATFA (H)	Forward: ATGCCGCCTTTGAAAAGTCCA	qPCR
	Reverse: TCTTCCGTTTGAGATAGTCCTCT	
MATFB (H)	Forward: ATGGATCACACAGGGGCGATA	qPCR
	Reverse: CCGCTGGGCTCTTCAAAGG	
KLF4 (H)	Forward: CCCACATGAAGCGACTTCCC	qPCR
	Reverse: CAGGTCCAGGAGATCGTTGAA	
FOXO1 (H)	Forward: TCGTCATAATCTGTCCCTACACA	qPCR
	Reverse: CGGCTTCGGCTCTTAGCAAA	
FOXO3 (H)	Forward: CGGACAAACGGCTCACTCT	qPCR
	Reverse: GGACCCGCATGAATCGACTAT	
FOXO4 (H)	Forward: GGCTGCCGCGATCATAGAC	qPCR
	Reverse: GGCTGGTTAGCGATCTCTGG	
GAPDH (H)	Forward: GGAGCGAGATCCCTCCAAAAT	qPCR
	Reverse: GGCTGTTGTCATACTTCTCATGG	
Sirt7 (M)	Forward: GCACTTGGTTGTCTACACGG	qPCR
	Reverse: TGTCCATACTCCATTAGGACCC	
Myh11 (M)	Forward: ATGAGGTGGTCGTGGAGTTG	qPCR
	Reverse: GCCTGAGAAGTATCGCTCCC	
Acta2 (M)	Forward: CCCAGACATCAGGGAGTAATGG	qPCR
	Reverse: TCTATCGGATACTTCAGCGTCA	

Cnn1 (M)	Forward: TCTGCACATTTTAACCGAGGTC	qPCR
	Reverse: GCCAGCTTGTTCTTTACTTCAGC	
Tagln (M)	Forward: CCAACAAGGGTCCATCCTACG	qPCR
	Reverse: ATCTGGGCGGCCTACATCA	
Srf (M)	Forward: GGCCGCGTGAAGATCAAGAT	qPCR
	Reverse: CACATGGCCTGTCTCACTGG	
Myocd (M)	Forward: AGGAAGTTCCGATCAGTCTTACA	qPCR
	Reverse: GGTATTAAGCCTTGGTTAGCCAG	
Matfa (M)	Forward: AAGGAGGCTATCATTGTGGGC	qPCR
	Reverse: ACTGACTCGGGACTCCAAGG	
Matfb (M)	Forward: CCAAGTCCTCAAAGTGAGTCTG	qPCR
	Reverse: CTGGCAGGTTCTAAATGCAGC	
Klf4 (M)	Forward: GGCGAGTCTGACATGGCTG	qPCR
	Reverse: GCTGGACGCAGTGTCTTCTC	
Foxo1 (M)	Forward: CCCAGGCCGGAGTTTAACC	qPCR
	Reverse: GTTGCTCATAAAGTCGGTGCT	
Foxo3 (M)	Forward: GGGGAACCTGTCCTATGCC	qPCR
	Reverse: TCATTCTGAACGCGCATGAAG	
Foxo4 (M)	Forward: CTTCCTCGACCAGACCTCG	qPCR
	Reverse: ACAGGATCGGTTCGGAGTGT	
Gapdh (M)	Forward: AGGTCGGTGTGAACGGATTTG	qPCR
	Reverse: GGGGTCGTTGATGGCAACA	
Myh11 (R)	Forward: TAGCAGATCTGGGACCACCA	qPCR
	Reverse: CGAAGCCCTGCTTCTCTGAA	
Acta2 (R)	Forward: GGATCAGCGCCTTCAGTTCT	qPCR
	Reverse: CAGGGCTAGAAGGGTAGCAC	
Cnn1 (R)	Forward: CGGCGTCACCTCTATGATCC	qPCR
	Reverse: TCAAAGATCTGCCGCTTGGT	
Tagln (R)	Forward: GTTCTTAGCCCGGACAGCTT	qPCR
	Reverse: AGGATGGGGGAAACGGTAGA	
Gapdh (R)	Forward: TGTGAACGGATTTGGCCGTA	qPCR
	Reverse: GATGGTGATGGGTTTCCCGT	
MYH11 promoter (H)	Forward: GAGCAGGAAGGCCACTCG	CHIP qPCR
	Reverse: CGCGGACTCCTTCCTGCA	
ACTA2 promoter (H)	Forward: GTGGAATGCAGTGGAAGAGACC	CHIP qPCR
	Reverse: GACTTGAGCCTGTCGTTCAGTC	
CNN1 promoter (H)	Forward: CCATGAGGCAGGCACTATCG	CHIP qPCR
	Reverse: TCTCCAGATACCAACAGTCTATAGC	

TAGLN promoter (H)	Forward: CCGCTCCATCTCCAAAGCAT	CHIP qPCR
	Reverse: CAGTGACTCCACACAGGCTC	
Myh11 promoter (M)	Forward: AGCACTGGGGTCCCTATACC	CHIP qPCR
	Reverse: GGAATTCCCAGCGTGGCATA	
Acta2 promoter (M)	Forward: ACAGCTGCTGCCATTCACC	CHIP qPCR
	Reverse: AGCCAAGGTTCTGATTCCATTCAC	
Cnn1 promoter (M)	Forward: CTTGTGAGGCAAGCACAGTTG	CHIP qPCR
	Reverse: TGACAGACCTCCATTCACCAACC	
Tagln promoter (M)	Forward: AGTCCGGGTAACAAGGAAGG	CHIP qPCR
	Reverse: GGAGACATTCTCTGCATGCTT	
ApoE$^{-/-}$ (M)	Forward: GACTGGTTTCAAACTGTGGCTC	Genotyping PCR
	Reverse1: GGCTGGATATGGATGTTGTTGC	
	Reverse2: GTCTGTTTACCTCCCCTTGCC	
Sirt7$^{-/-}$ (M)	Forward: CTCGATGTATTGGAATTACCTGCC	Genotyping PCR
	Reverse1: ACTTGTATGGATAGGGGCAGATGG	
	Reverse2: CCCGATAATCTGGGATAGAAGCTG	
Sirt7$^{flox/flox}$ (M)	Forward: CAGGATTTAAGTGTGTGGACAGAG	Genotyping PCR
	Reverse: CCACATACCAAACAGAGAACCCTA	
Tagln-Cre (M)	Forward: CAGACACCGAAGCTACTCTCCTTCC	Genotyping PCR
	Reverse: CGCATAACCAGTGAAACAGCATTGC	
shSIRT7 (H)	GTCCAGCCTGAAGGTTCTAAA	Knockdown
shSRF (H)	GAGACCGGCAAGGCACTGATT	Knockdown
shXPO1 (H)	CCATTGTAAAGCGACTTCAAA	Knockdown
shXPO4 (H)	CCCTAGAATTAGGAATGACAT	Knockdown
shXPO5 (H)	CCAGATGTTTCGAACACTAAA	Knockdown
shXPO7 (H)	CGTGAATAGCAATGACATGAT	Knockdown
shSirt7 (M)	TGCATCCCTAACAGAGAGTAT	Knockdown
shXpo1 (M)	CCCATTGTAAAGCAACTTCAA	Knockdown
shXpo4 (M)	GCTACCTCTTAGCTGATGATA	Knockdown
shXpo5 (M)	GAACATGGAAAGAATCTTGAA	Knockdown
shXpo7 (M)	CGACAGAGAATTGAGCCATTT	Knockdown
shSirt7 (R)	GGTCCTGTGTCACAGTGAA	Knockdown
sgSirt7 (R)	Seq1: GCCGGACGTGCCACAAGTGT	Knockout
	Seq2: GCTTGAGGCCCTCACGCCGC	

Supplementary Data 1. (separate file)

Systematic mass spectrometry analysis to identify potential substrate proteins: HASMC cells were infected with Flag-SIRT7 or empty vector control lentivirus, and then pulled down Flag-SIRT7 using anti-Flag antibody magnetic beads.

Supplementary Data 2. (separate file)

SRF post-transcriptinal modifications (Acetylations and phosphorylations)

Supplementary Fig. 1

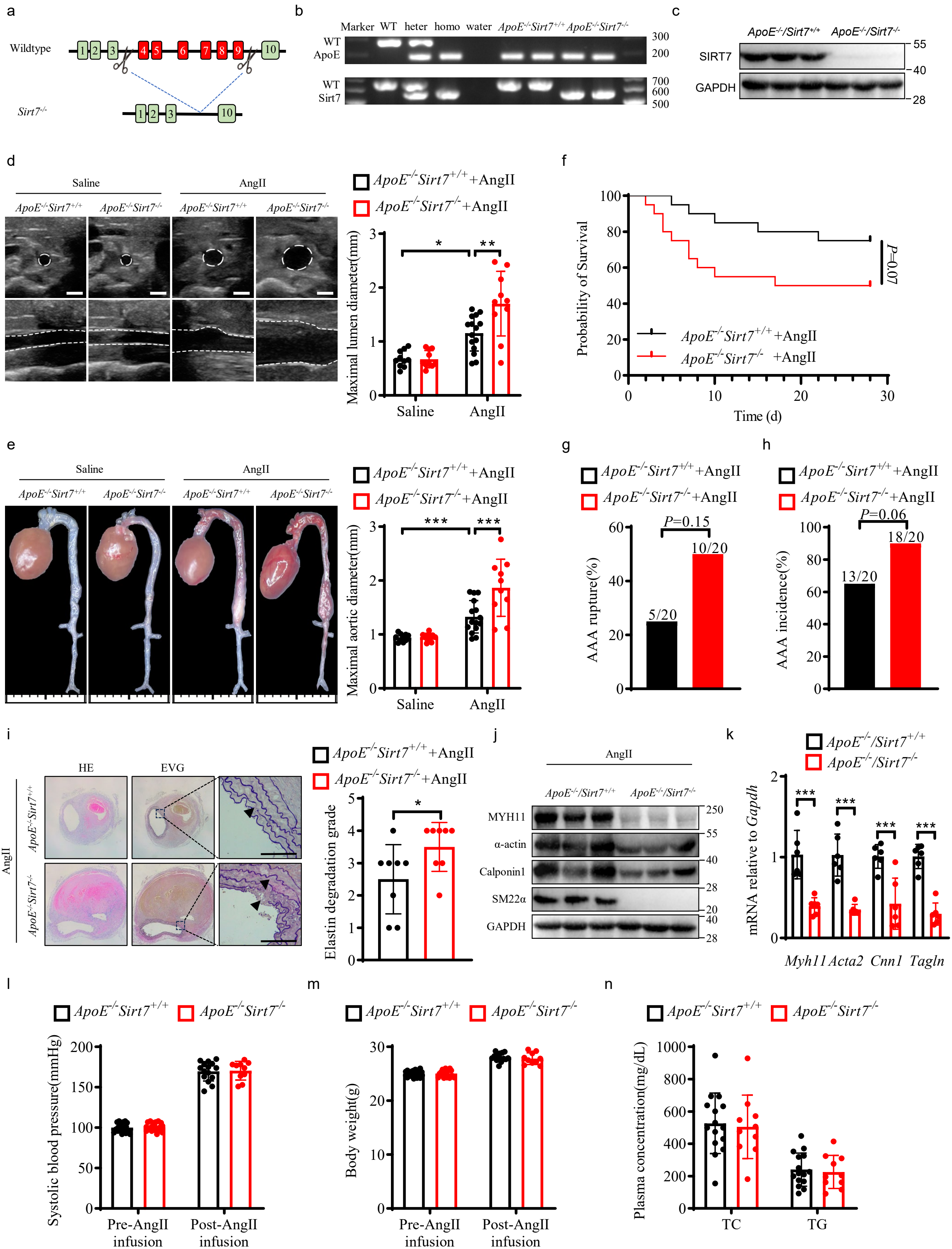

Supplementary Fig. 2

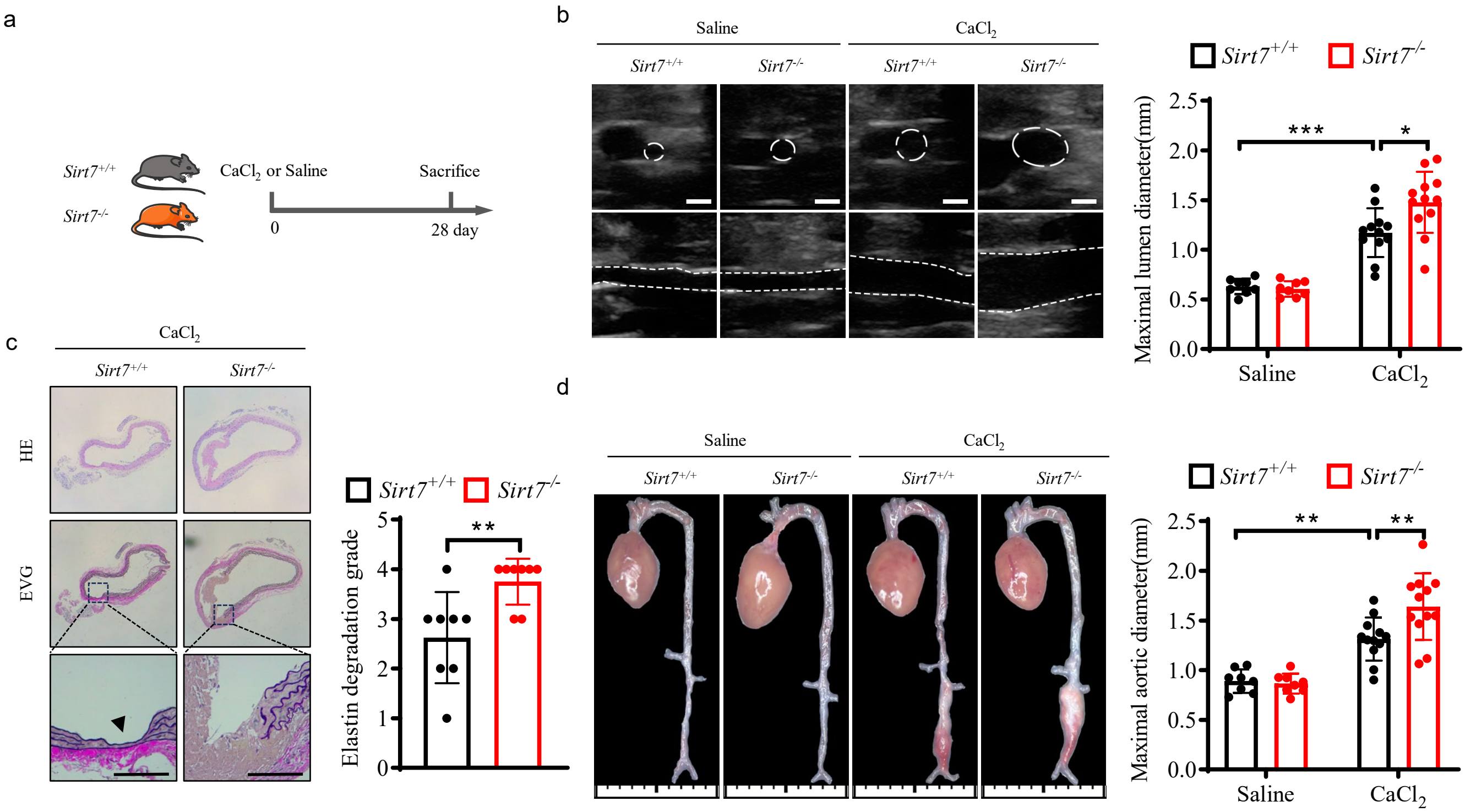

Supplementary Fig. 3

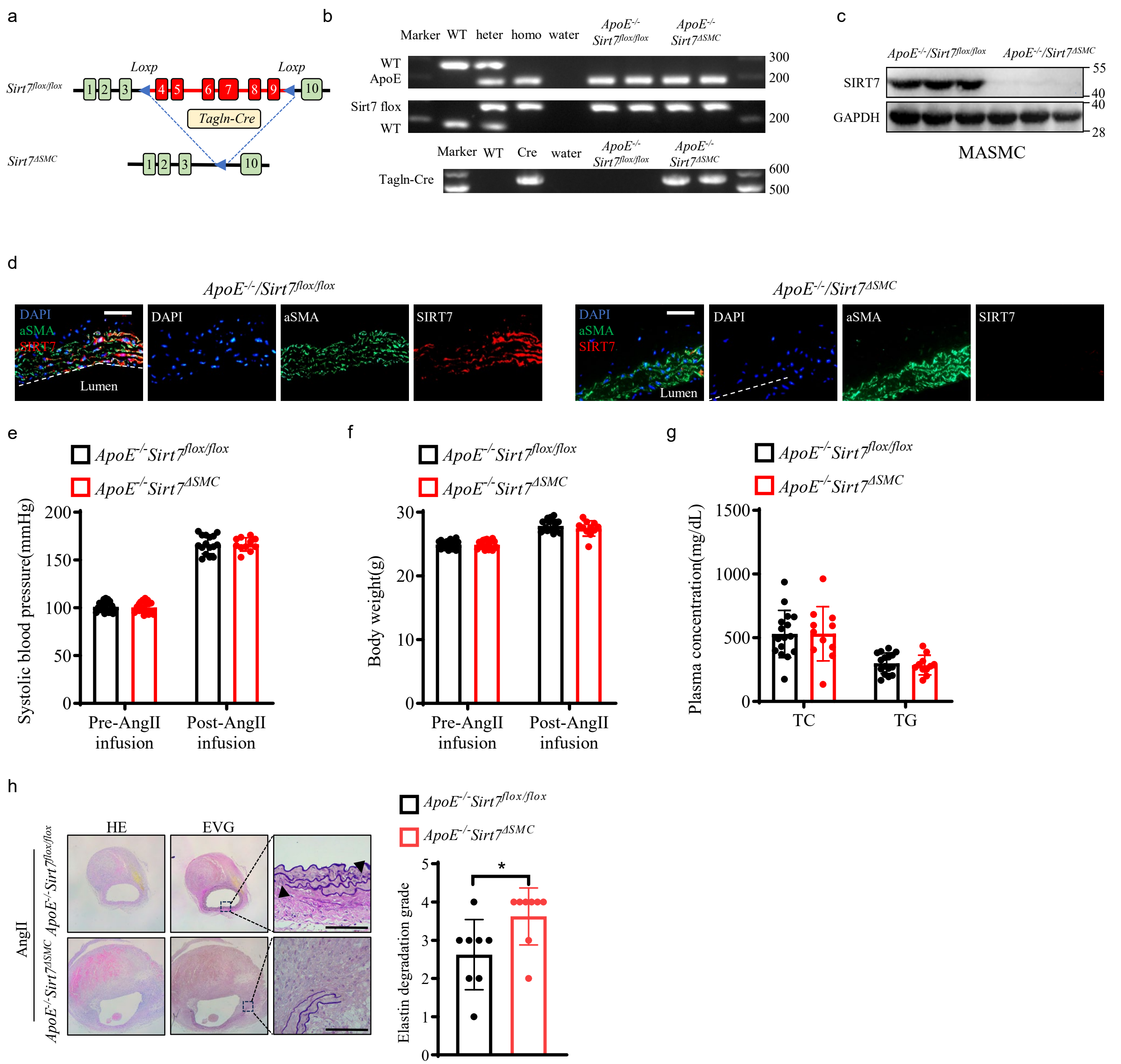

Supplementary Fig. 4

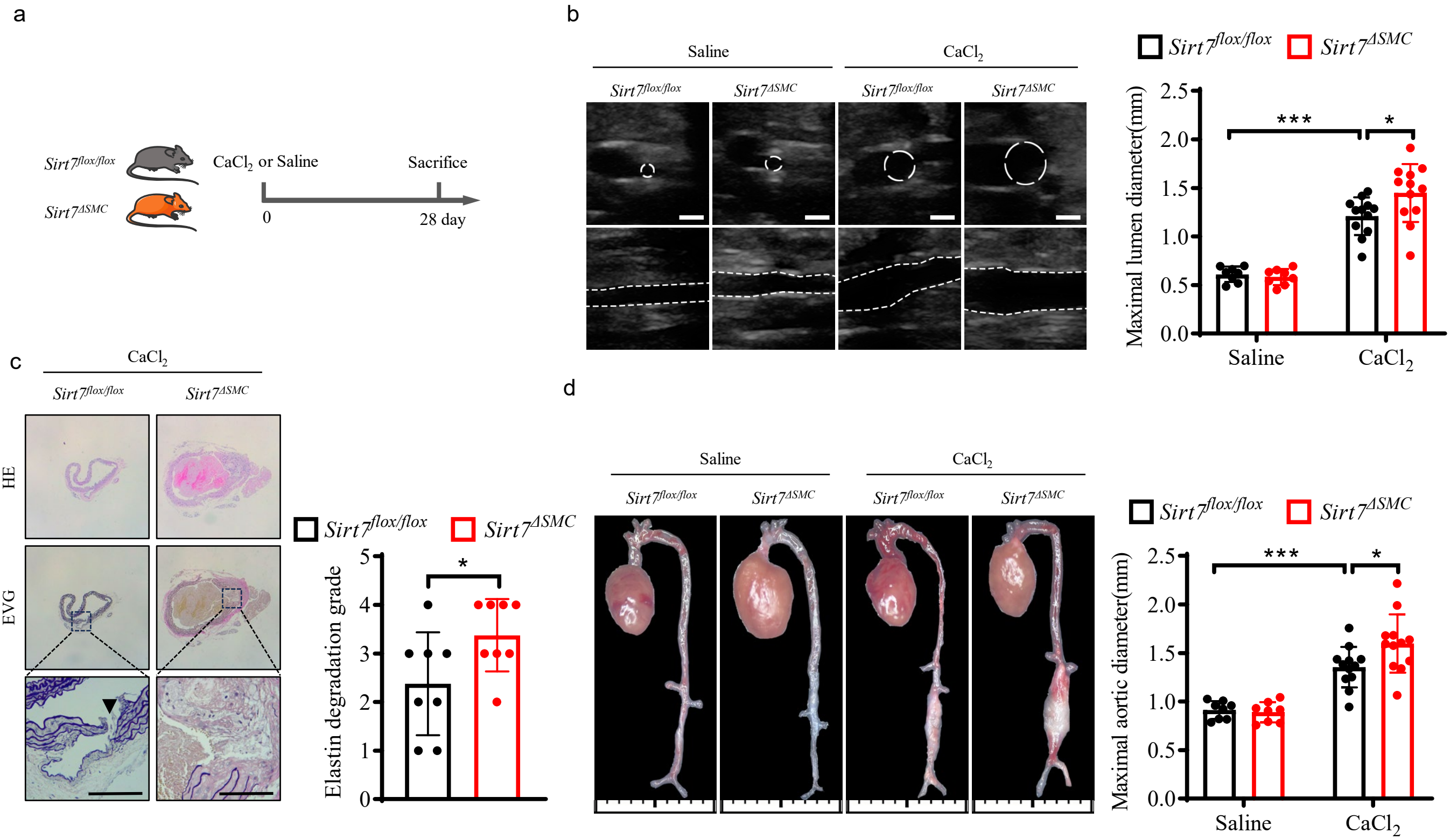

Supplementary Fig. 5

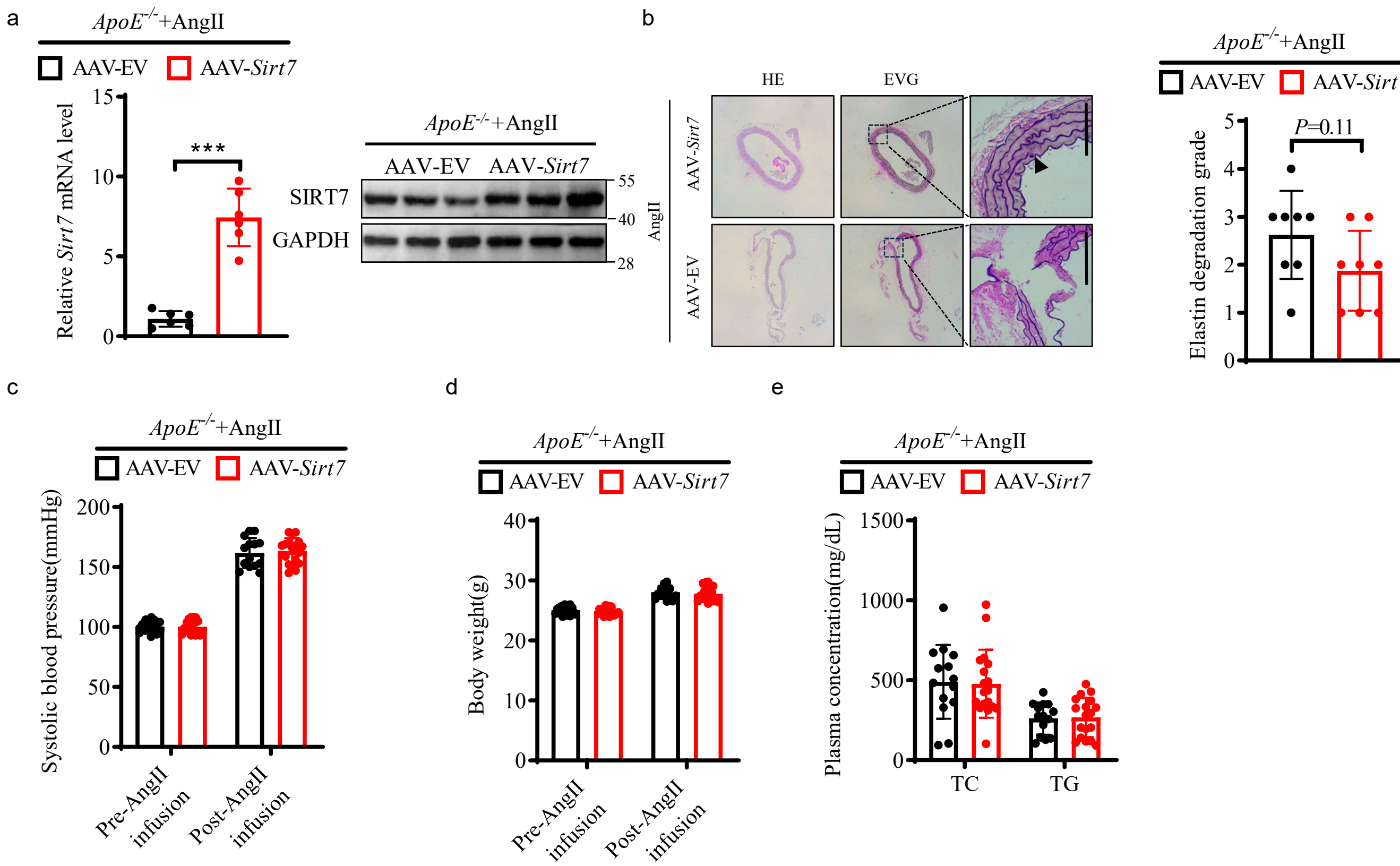

Supplementary Fig. 6

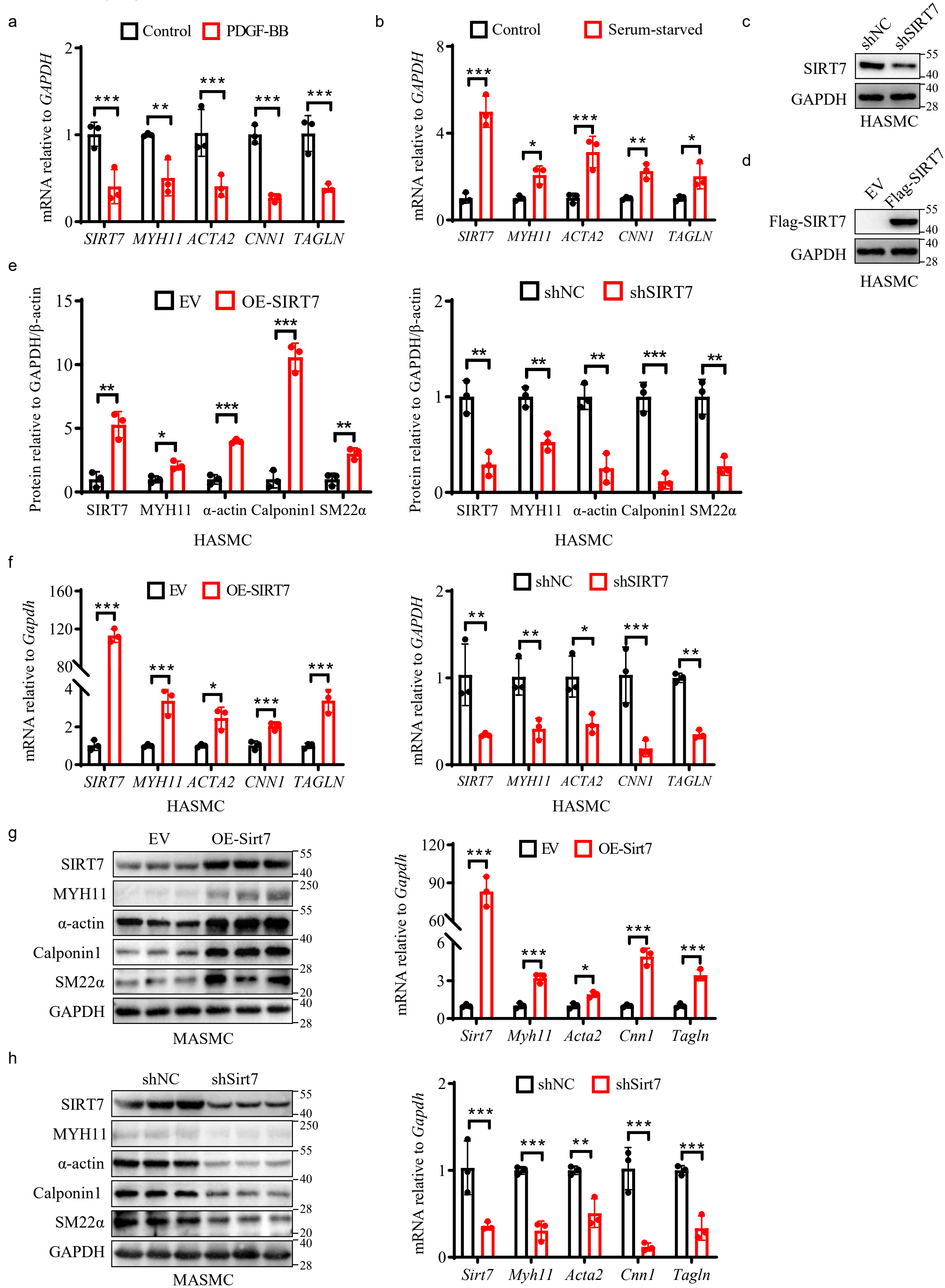

Supplementary Fig. 7

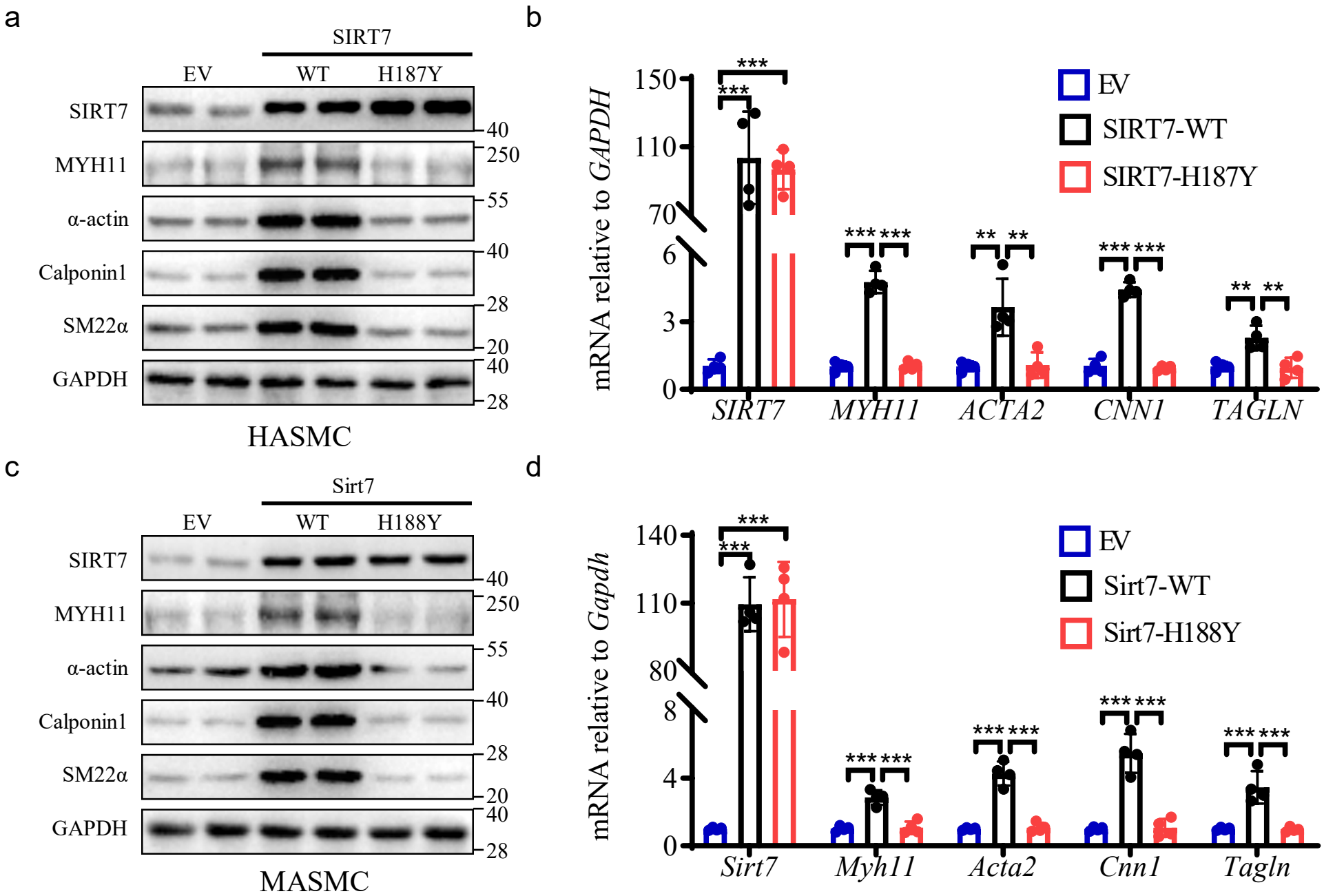

Supplementary Fig. 8

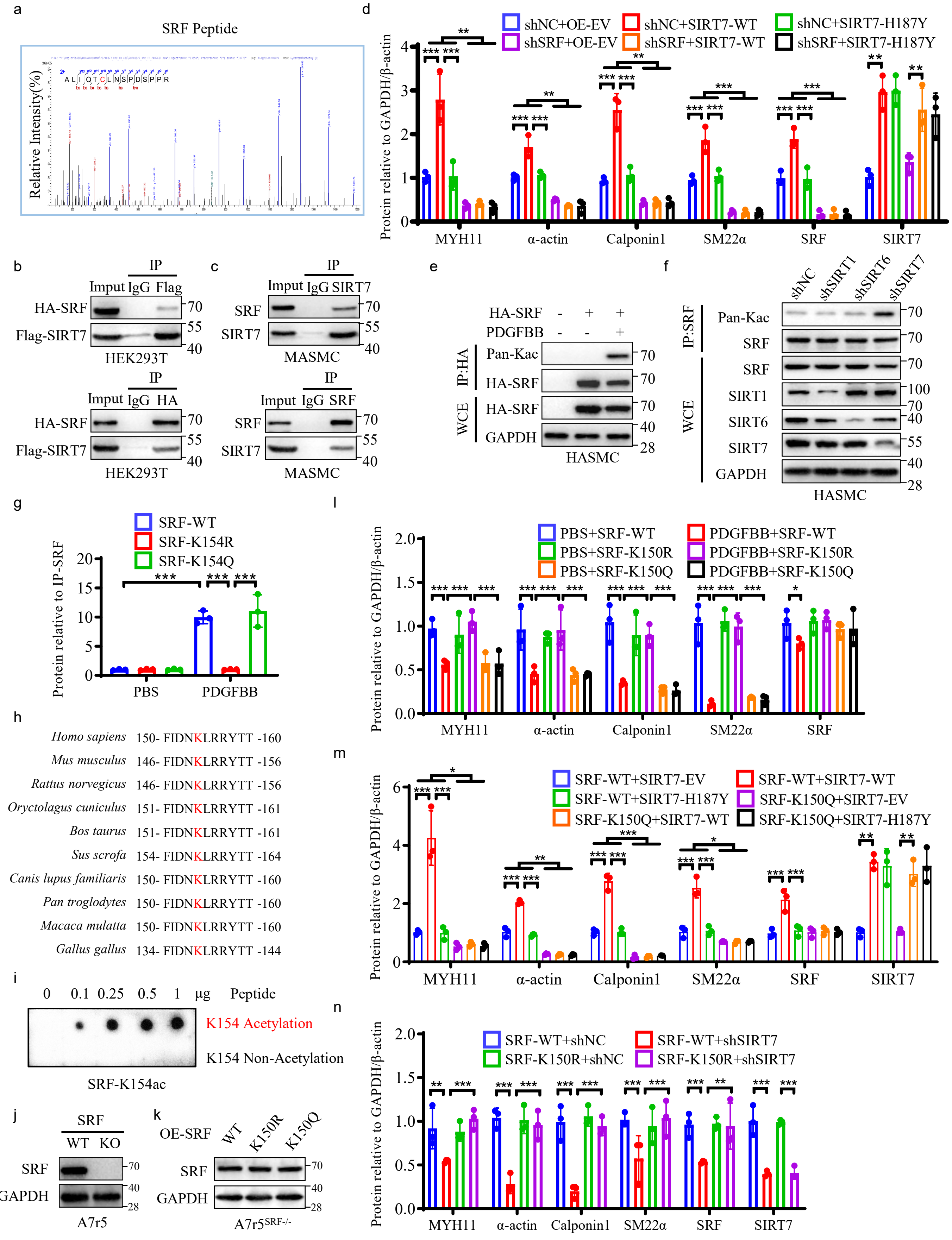

Supplementary Fig. 9

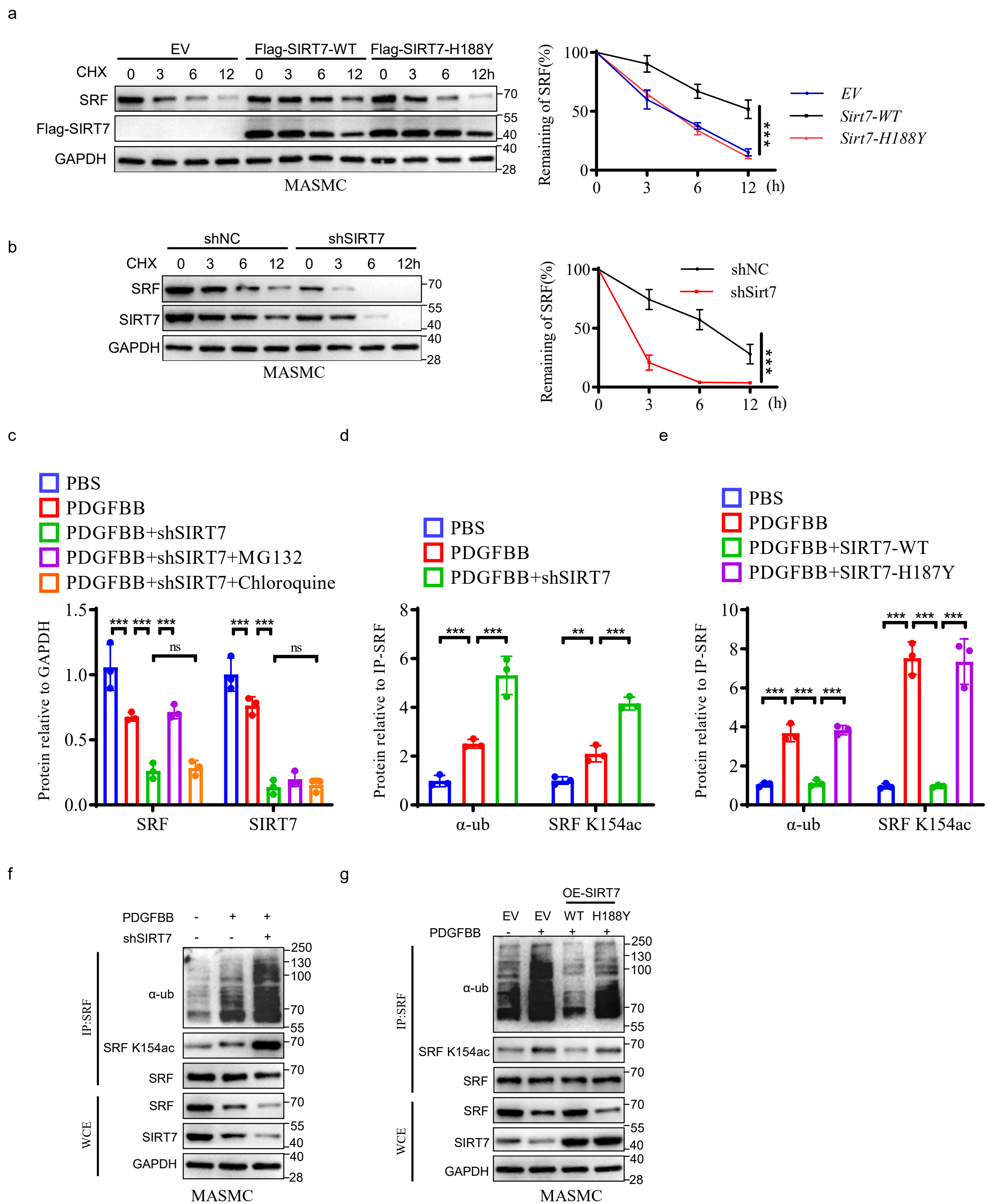

Supplementary Fig. 10

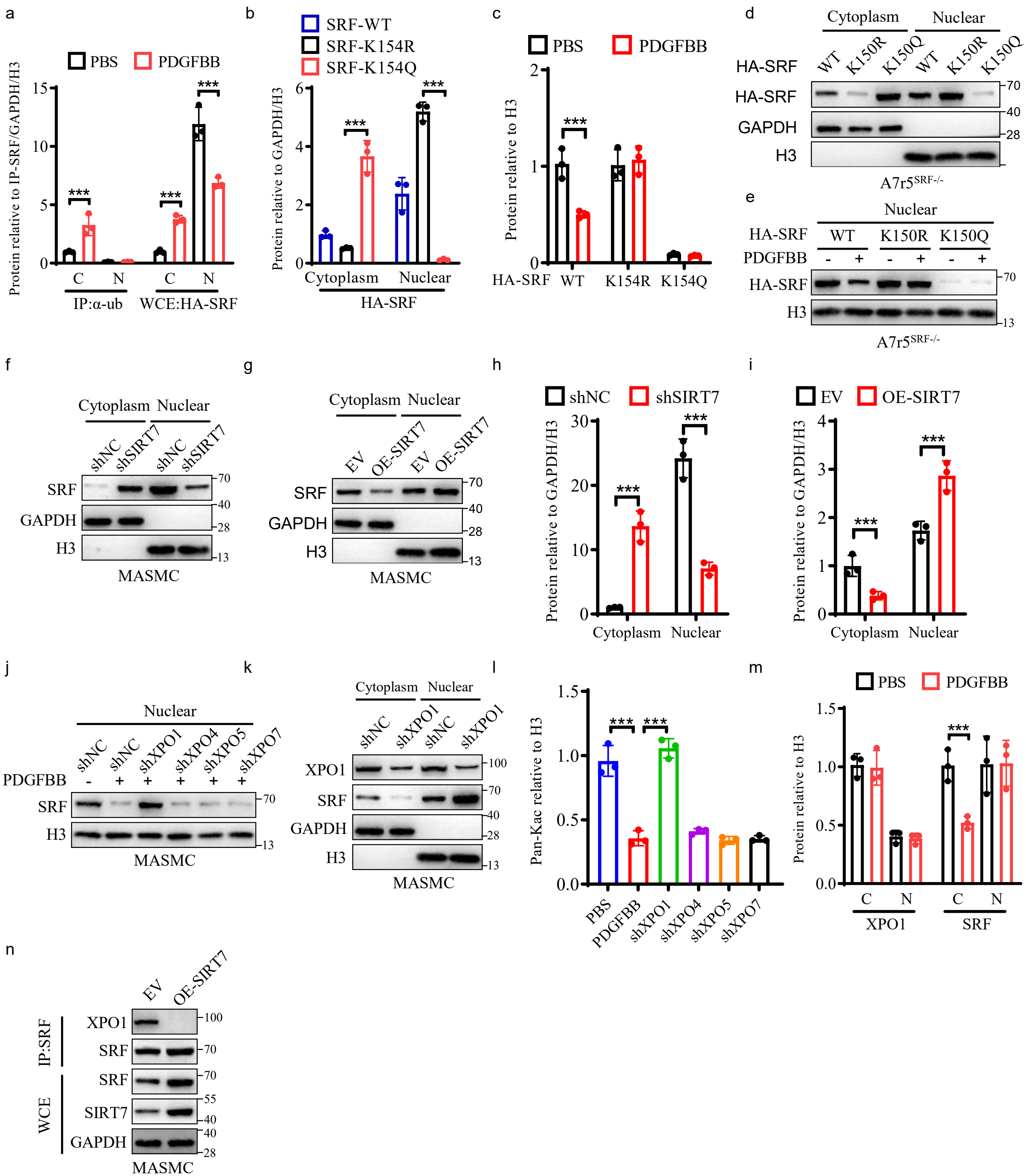

Supplementary Fig. 11

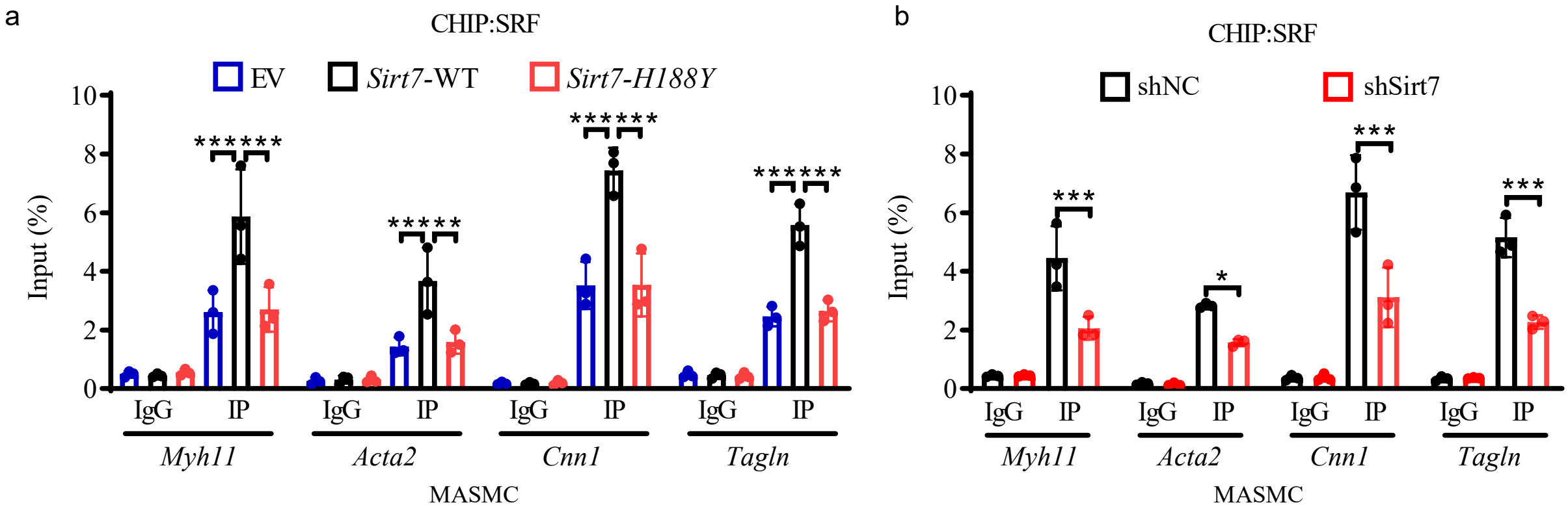

Supplementary Fig. 12

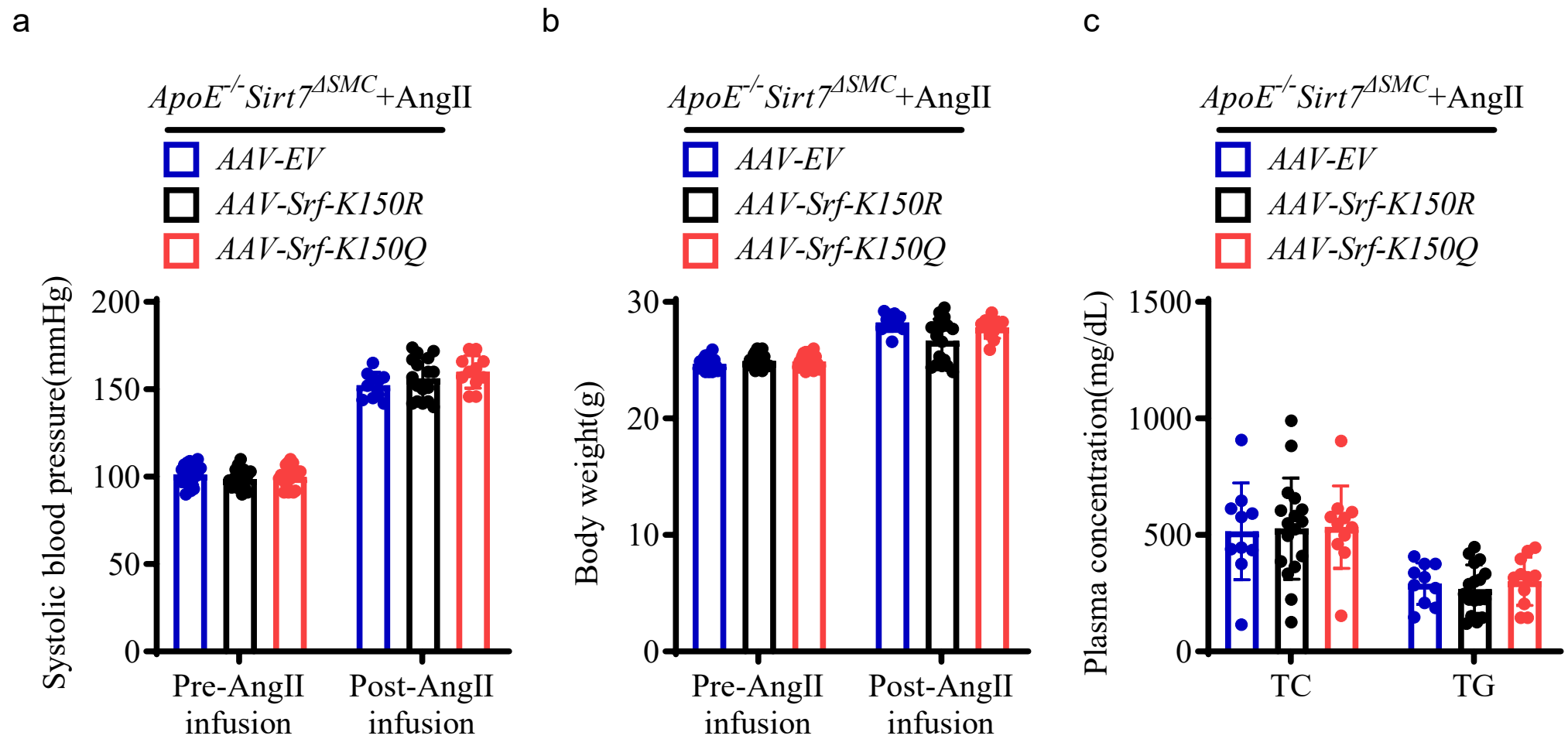

Supplementary Fig. 13

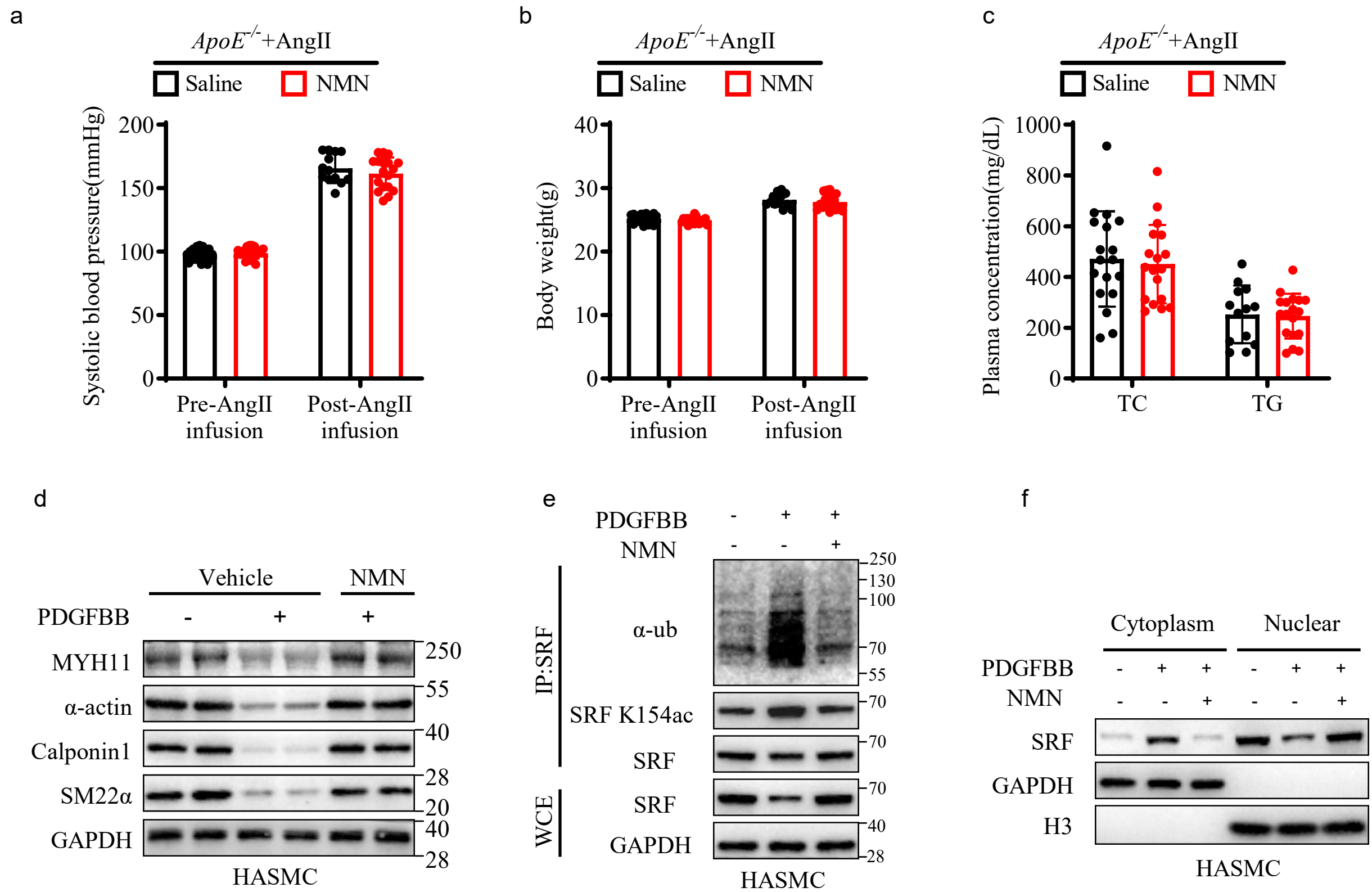

Supplementary Fig. 14

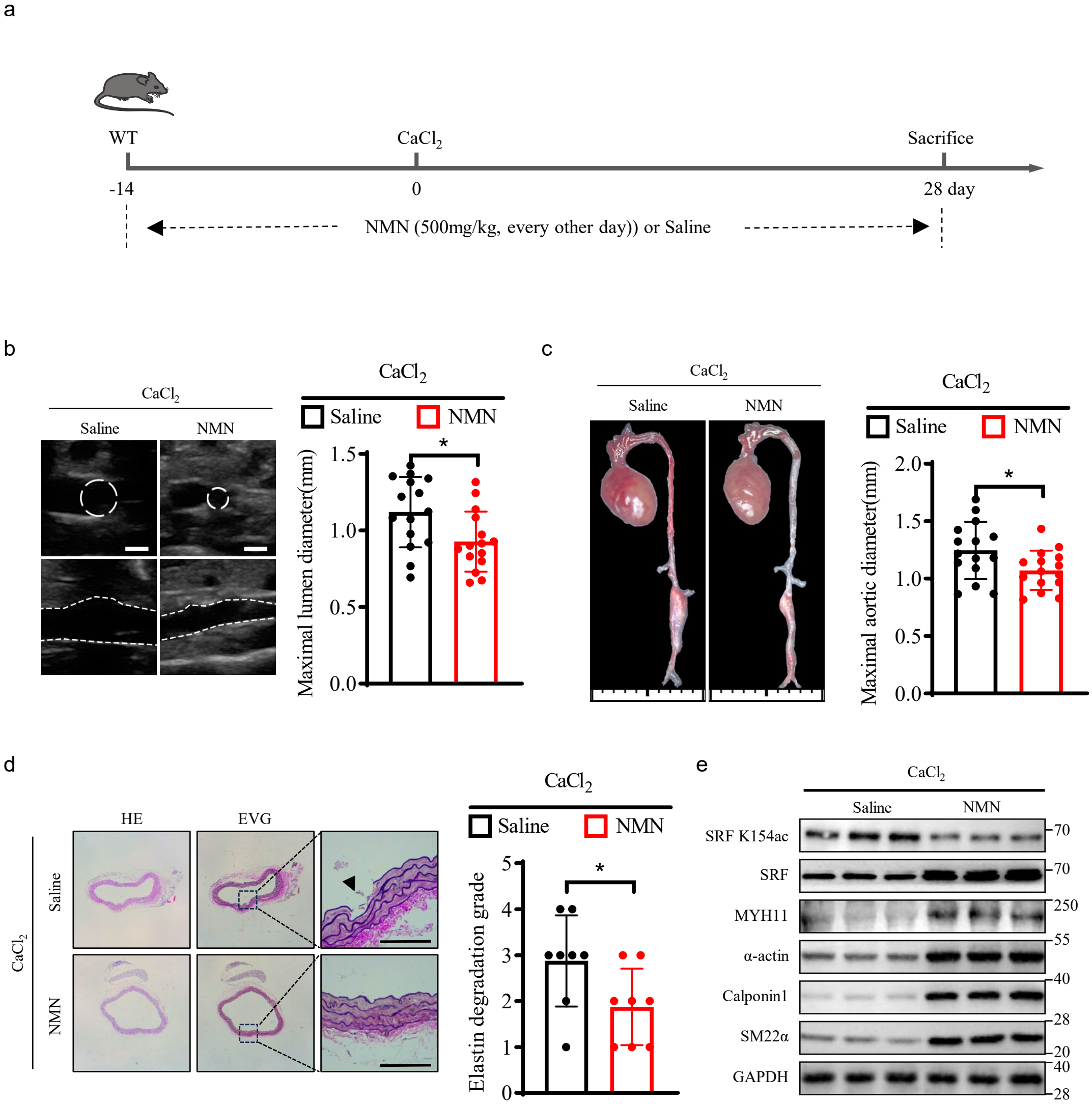

Supplementary Fig. 15

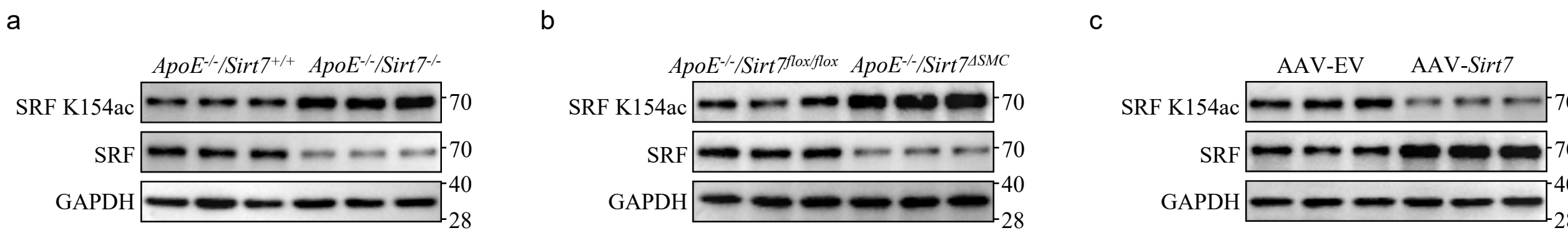

Supplement: Supplementary file 1 [file 12276_2026_1787_MOESM1_ESM.pdf]
